# Droplet digital PCR assay provides intrahepatic HBV cccDNA quantification tool for clinical application

**DOI:** 10.1038/s41598-022-05882-9

**Published:** 2022-02-08

**Authors:** Sanae Hayashi, Masanori Isogawa, Keigo Kawashima, Kyoko Ito, Natthaya Chuaypen, Yuji Morine, Mitsuo Shimada, Nobuyo Higashi-Kuwata, Takehisa Watanabe, Pisit Tangkijvanich, Hiroaki Mitsuya, Yasuhito Tanaka

**Affiliations:** 1 Department of Virology and Liver Unit, Nagoya City University Graduate School of Medical Sciences, Nagoya, Japan; 2 Center of Excellence in Hepatitis and Liver Cancer, Faculty of Medicine, Chulalongkorn University, Bangkok, Thailand; 3 Department of Surgery, Tokushima University, Tokushima, Japan; 4 Department of Refractory Viral Infections, National Center for Global Health and Medicine Research Institute, Tokyo, Japan; 5 Experimental Retrovirology Section, HIV and AIDS Malignancy Branch, National Cancer Institute, National Institutes of Health, Bethesda, MD USA; 6 Department of Clinical Sciences, Kumamoto University Hospital, Kumamoto, Japan; 7 Department of Gastroenterology and Hepatology, Faculty of Life Sciences, Kumamoto University, 1-1-1 Honjo, Chuo-ku, Kumamoto, 860-8556 Japan

**Keywords:** Hepatology, Hepatitis B

## Abstract

The persistence of covalently closed circular DNA (cccDNA) poses a major obstacle to curing chronic hepatitis B (CHB). Here, we used droplet digital PCR (ddPCR) for cccDNA quantitation. The cccDNA-specific ddPCR showed high accuracy with the dynamic range of cccDNA detection from 10^1^ to 10^5^ copies/assay. The ddPCR had higher sensitivity, specificity and precisely than qPCR. The results of ddPCR correlated closely with serum HB core-related antigen and HB surface antigen (HBsAg) in 24 HBV-infected human-liver-chimeric mice (PXB-mice). We demonstrated that in 2 PXB-mice after entecavir treatment, the total cccDNA content did not change during liver repopulation, although the cccDNA content per hepatocyte was reduced after the treatment. In the 6 patients with HBV-related hepatocellular carcinoma, ddPCR detected cccDNA in both tumor and non-tumor tissues. In 13 HBeAg-negative CHB patients with pegylated interferon alpha-2a, cccDNA contents from paired biopsies were more significantly reduced in virological response (VR) than in non-VR at week 48 (p = 0.0051). Interestingly, cccDNA levels were the lowest in VR with HBsAg clearance but remained detectable after the treatment. Collectively, ddPCR revealed that cccDNA content is stable during hepatocyte proliferation and persists at quantifiable levels, even after serum HBsAg clearance.

## Introduction

Approximately 257 million people are estimated to be chronically infected with hepatitis B virus (HBV) worldwide and 887,000 deaths every year are attributed to sequelae of HBV infection^1^. Although nucleos(t)ide analogs (NAs) inhibiting viral DNA synthesis are approved antiviral therapies for chronic HBV (CHB) infection^2,3^, they cannot eliminate the virus due to the persistence of covalently closed circular DNA (cccDNA) in the hepatocytes.

Currently, intrahepatic cccDNA is detected by quantitative real-time PCR (qPCR) or Southern blotting (SB). However, the amount of cccDNA in human hepatocytes is rather low, limiting the use of SB^4,5^. Because cccDNA is very stable, its reduction may be slow. Therefore, a more precise measurement is desirable to evaluate the impact of new anti-HBV drugs on cccDNA. Droplet digital PCR (ddPCR) provides an accurate copy number of a single gene sequence^4,5^. Several studies have been systematically compared the quantitative accuracies of ddPCR and qPCR in detecting intrahepatic cccDNA, but the kinetic analysis of cccDNA using ddPCR and its clinical application is limited. Meanwhile, clinical studies revealed the potential of serum hepatitis B surface antigen (HBsAg) and hepatitis B core-related antigen (HBcrAg) levels as surrogate biomarkers for intrahepatic cccDNA in CHB patients, but little information is available regarding the extent to which HBsAg marker reflects the cccDNA content in the liver in the absence of antibody response to HBsAg under immune suppression, as well as the possibility of HBsAg synthesized from integrated HBV DNA needs to be considered^6–9^. Moreover, the impact of cell division on intrahepatic cccDNA content remains contentious, partly due to the lack of a highly quantitative methodology.

Here, we developed a new assay to quantitate cccDNA content using ddPCR. This ddPCR assay could measure cccDNA more accurately and with greater specificity and sensitivity than qPCR. The amount of cccDNA was stable during hepatocyte proliferation, and the amount of cccDNA was shown to be reduced under PEG-IFN treatment in vivo.

## Materials and methods

### Ethics statement

This study was performed in accordance with the relevant national guidelines and regulations.

### Chimeric mice with human hepatocytes

Severe combined immunodeficiency mice transgenic for the urokinase-type plasminogen activator gene (cDNA-uPA^wild/+^/SCID+/+ mice), with their livers replaced by human hepatocytes, (human-liver-chimeric mice) were purchased from PhoenixBio Co., Ltd. (Hiroshima, Japan). These mice were infected with sera obtained from human hepatocyte chimeric mice previously infected with HBV subgenotype C2/Ce, as described in a previous report^10^. 24 HBV-infected chimeric mice were under various conditions at the time of autopsy, and seven mice showed low HBV infection efficacy due to ETV treatment during the acute phase of infection (Table S2). The in-life phase of this study was outsourced to PHOENIXBIO Co., Ltd. The protocol of this study was reviewed by Ethics Committee of PHOENIXBIO, approved on June 15, 2017 (Resolution No.: 1910) prior to the start of the in-life phase on June 16, 2017 and carried out in compliance with the ARRIVE guidelines. More detailed procedures are given in the Supplementary Information.

### Patients

A total of 6 HCC patients and 13 hepatitis B e antigen (HBeAg)-negative patients with CHB were enrolled in this study. Liver tumor tissues and the corresponding non-tumor tissues were collected from five patients with HBV infection (3 HBsAg-positive and 2 HBsAg-negative/anti-HBc-positive) and one patient without HBV infection (HBsAg-negative and anti-HBc-negative) at Tokushima University Hospital and Nagoya City University Hospital in Japan. Thirteen HBeAg-negative patients who received 48 weeks of PEG-IFN-alfa2a (180 µg/week) monotherapy at the King Chulalongkorn Memorial Hospital, Bangkok, Thailand, were enrolled into this study, and paired liver tissues were collected before and after PEG-IFN treatment. Virological response (VR) was defined as an HBV DNA level below 2000 IU/mL at 48 weeks after treatment. Thirteen patients with HBeAg-negative CHB who received 48 weeks of PEG-IFN monotherapy were categorized into 3 groups: non-VR (n = 6), VR without HBsAg clearance (n = 4) and VR with HBsAg clearance (n = 3). At baseline, there was no significant difference in the levels of HBV DNA, HBsAg, or HBcrAg in the serum, or the levels of total cccDNA and cccDNA per cell in the liver, among the 3 groups (p = 0.26473, p = 0.64468, p = 0.69439, p = 0.30080, and p = 0.5565, respectively) (Table S3). This study was approved by the Institutional Review Boards at all three participating institutes: Nagoya City University Graduate School of Medical Sciences and Nagoya City University Hospital Institutional Review Board, Ethics Committee of Tokushima University Hospital and the faculty of Medicine, Chulalongkorn University. Written informed consent was obtained from each patient and the study protocol conformed to the ethical guidelines of the Declaration of Helsinki and was approved by the appropriate institutional ethics review committees of each institute. More detailed procedures are given in the Supplementary Information.

### Analysis of virological markers

Serum HBV DNA titers were measured in chimeric mice by real-time PCR, as described previously^11,12^. Hepatitis B surface antigen (HBsAg) and hepatitis B core-related antigen (HBcrAg) were measured by chemiluminescent enzyme immunoassays using commercial kits (FUJIREBIO Inc., Tokyo, Japan), as described previously^10,13^. The detection limits of the HBsAg and HBcrAg assays are 0.005 IU/ml and 3 logU/ml, respectively. The HBV DNA titers were measured in patients using the Abbott Real Time HBV assay or the TaqMan polymerase chain reaction assay (COBAS TaqMan, ROCHE MOLECULAR SYSTEMS [lower detection limit: 20 IU/ml]) or Abbott RealTime HBV assay (lower detection limit: 10 IU/ml). More detailed procedures are given in the Supplementary Information.

### Isolation of genomic DNA from the livers of chimeric mice

Genomic DNA was isolated from the livers of chimeric mice using the phenol/chloroform method, as described previously^14^. Protein-free DNA extraction from the mouse livers was carried out using a modified Hirt extraction procedure^15^. The genomic DNA and protein-free DNA were digested with DNase-free RNase and restriction enzyme (*Hind* III) before analysis. More detailed procedures are given in the Supplementary Information.

### Quantitation of HBV cccDNA and relaxed circular (rc) DNA by ddPCR

**Figure 1:**
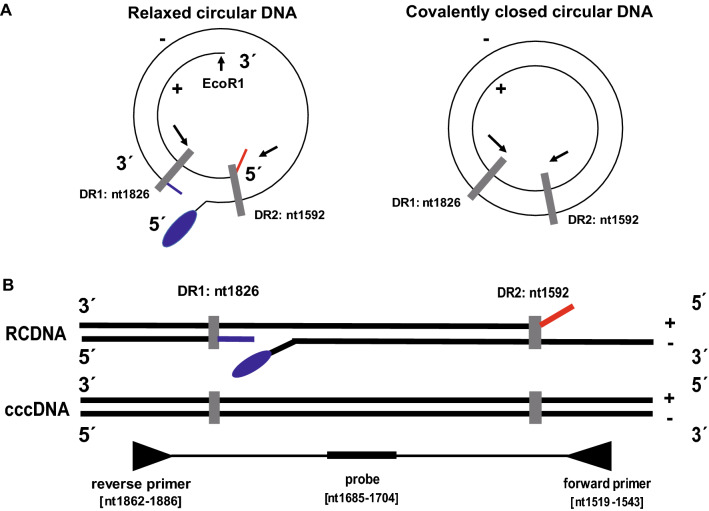
The structure of the HBV genome. (**A**) The HBV relaxed circular DNA (rcDNA) and cccDNA. There are two direct repeats (DRs) at nucleotides (nt) 1826 and 1592, and the numbering starts at the Eco RI site. rcDNA has a complete minus strand with a nine-base terminal redundancy (blue line) and a terminal protein polymerase attached to the 5′-end (blue oval). The plus strand has a defined 5′-end with an RNA primer (red line) but a variable 3′-end. PCR primers for amplification of cccDNA are targeted to opposite sides of the single-stranded gap regions of rcDNA. (**B**) The cccDNA-specific forward and reverse primers are 5′-ACGGGGCGCACCTCTCTTTACGCGG-3′ [nt 1519–1543] and 5′-CAAGGCACAGCTTGGAGGCTTGAAC-3′ [nt 1862–1886], respectively, and the probe: 5′-FAM-AACGACCGACCTTGAGGCAT-MGB-3′.

Total liver DNA treated with plasmid-safe ATP-dependent DNase (PSAD) (EPICENTRE, Madison, Wisconsin, USA) was used as template inputs for either qPCR or ddPCR amplification. The cccDNA specific primers-probe set for cccDNA amplification designed as shown in Fig. 1A, B was used for both ddPCR and qPCR assays. After generating reaction droplets, intrahepatic cccDNA was amplified using a C1000 touch Thermal Cycler (BIO-RAD LABORATORIES, Hercules, California, USA). In some cases, intrahepatic cccDNA values were normalized by the cell number measured by the hRPP30 copy number variation assay (BIO-RAD LABORATORIES, Pleasanton, California, USA)^16^. Of note, hRPP30 levels were separately determined using DNA that was not treated with PSAD, which digests genomic DNA. Since the amount of DNA genome diluted to various concentrations in the range of 125–1000 ng/assay in a vial accurately measured the number of cccDNA (R^2^ value greater than 0.99), up to 1000 ng of total DNA per vial was measured in HCC case. More detailed procedures are given in the Supplementary Information.

### Quantitation of HBV cccDNA by qPCR

Target intrahepatic cccDNA was amplified by the StepOnePlus Real-Time PCR System (THERMO FISHER SCIENTIFIC, Waltham, MA). Serial dilutions of a plasmid containing an HBV monomer (pHBV: AB246337) served as quantitation standards. More detailed procedures are given in the Supplementary Information.

### Detection of HBV cccDNA by southern blotting

Genomic DNA and protein-free DNA was isolated from the livers of chimeric mice infected with HBV. The DNAs were digested with the restriction enzyme, *Hind* III*,* that does not cleave HBV, and analyzed by Southern blotting. The signals were analyzed using an ImageQuant LAS 4000mini (GE HEALTHCARE, United Kingdom). To confirm the nature of cccDNA, the samples were subsequently digested with *Xho* I to linearize the DNA. Agarose gel electrophoresis and Southern blotting were performed as described previously^17^.

### Statistical analysis

The coefficient of determination (R^2^) was assessed by linear regression analysis. The correlation between two continuous variables was analyzed using Spearman's rank correlation coefficient. The Mann–Whitney U-test was used to examine statistical difference between the VR and non-VR groups. The limits of quantitative value (LOQ) and detection value (LOD) for HBV-cccDNA-specific ddPCR were determined using serial dilutions of total DNA extracted from non-infected and HBV-infected livers (chimeric mice or human) using classical method. LoD and LoQ were defined as the lowest concentrations at which 50% and 95% of positive samples were detected, respectively. The LOD and LOQ in the mouse liver were 1 copy/ 20 ng assay and 20 copies/ 20 ng assay, respectively. The LOD and LOQ in the human liver were 1 copy/ 20 ng assay and 15.8 copies/20 ng assay, respectively. The Statistical analyses were performed using BellCurve for Excel (version 3.21) (SOCIAL SURVEY RESEARCH INFORMATION Co., Ltd., Tokyo, Japan).

## Results

### Optimization and validation of ddPCR for the detection of HBV cccDNA

In the HBV genome, the incomplete plus strand has a variable 3′-end but a defined 5′-end at the position of 1592 near DR2, while the complete minus strand has defined 5′- and 3′-ends with a terminal redundancy of 9 bases. There is a gap at the position of 1826 near DR1 (Fig. 1A, B). To discriminate between the relaxed circular DNA (rcDNA) and the cccDNA of the HBV genome, we designed the primer to bind the negative strand on one side of the gap, and the probe to bind the same strand on the other side. Therefore, the forward and reverse primers, 5′-ACGGGGCGCACCTCTCTTTACGCGG-3′ [nt: 1519–1543] and 5′-CAAGGCACAGCTTGGAGGCTTGAAC-3′ [nt: 1862–1886], were designed against the minus and plus strand gaps of rcDNA and selected to specifically amplify DNA fragments from replication intermediate cccDNA but not from viral genome DNA. A TaqMan probe, which binds to DNA when PCR is performed, is composed of FAM and MGB quencher (Fig. 1B). To establish an assay to detect HBV cccDNA using ddPCR technology, we used total DNA extracted from the livers of human-liver-chimeric mice that had been infected with HBV (Fig. 1). Digestion of DNA by a restriction enzyme is believed to improve template accessibility^18^. To test the effect of restriction enzyme digestion on the efficacy of cccDNA detection, temperature gradient ddPCR was performed in the presence of Hae III with fifteen restriction sites in the HBV genome outside the cccDNA amplification region on the sample DNA treated with PSAD. Xho I, which does not recognize the cccDNA amplification region but cuts at one site in the HBV genome, was used for comparison. As shown in Supplementary Fig. S1A ,B, Hae III digestion provided better separation of positive and negative signals, and the results were less affected by the temperature as the signal intensity of positive droplet populations started to decrease at the annealing temperature below 58.5 °C for Xho 1 and 55.3 °C for Hae III. Based on these results, we decided to use Hae III digestion and set the annealing temperature at 61.2 °C.

### Accuracy of cccDNA quantification by ddPCR and qPCR assays

**Figure 2:**
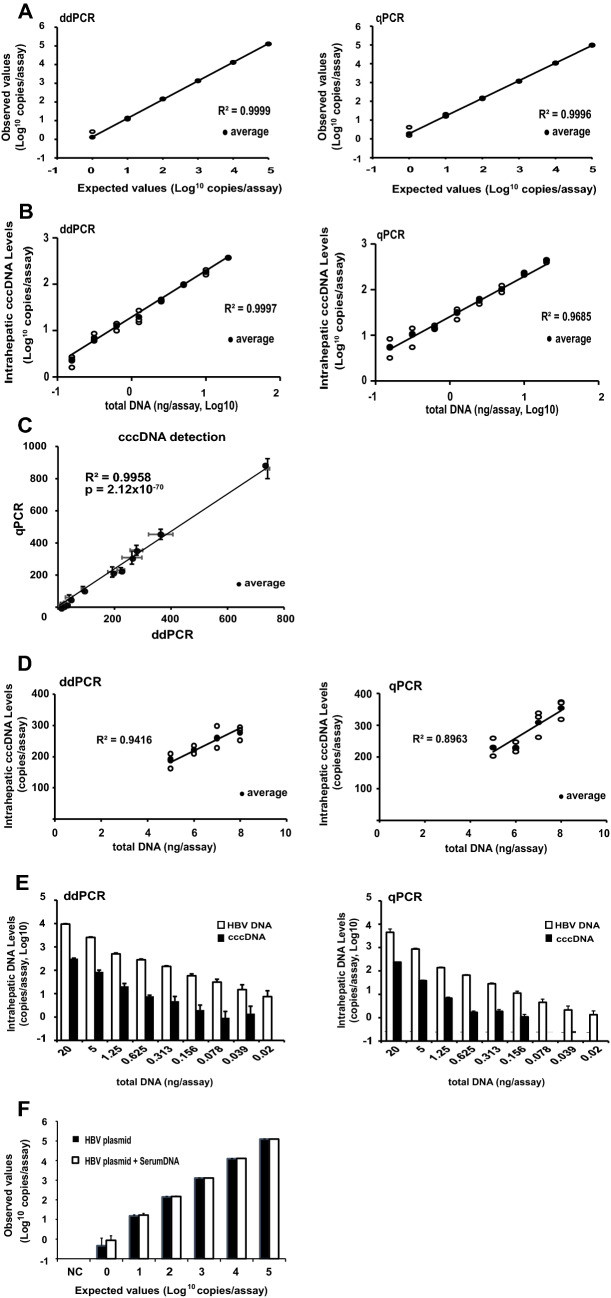
Comparison of ddPCR to real-time PCR for quantitation of cccDNA. (**A**) The plasmid containing the HBV genotype C2/Ce DNA genome (AB246345) was measured using a tenfold dilution of DNA ranging from 10^5^ to 10^0^ copies/assay by ddPCR or real-time PCR. (**B**) Intrahepatic cccDNA levels in the liver of chimeric mouse infected with HBV were measured using various amounts of DNA ranging from 20 to 0.156 ng/assay by ddPCR or real-time PCR. (**C**) The intrahepatic cccDNA levels measured by ddPCR and real-time PCR revealed substantial agreement. (**D**) Intrahepatic cccDNA levels from the liver of chimeric mouse with HBV infection were measured using various amounts of DNA, ranging from 8 to 5 ng/assay. (**E**) Intrahepatic HBV DNA and cccDNA levels in the liver of chimeric mouse infected with HBV were measured using various amounts of DNA ranging from 20 to 0.02 ng/assay by ddPCR or real-time PCR. Each assay was conducted in triplicate. Target concentrations were repeatedly and independently tested three times. (**F**) The comparison of the HBV plasmid using a tenfold dilution of DNA ranging from 10^5^ to 10^0^ copies/assay with or without the sera from IC patient (#9 in Fig. S3C) by ddPCR. The cccDNA signal in the absence of the HBV plasmid was negative irrespective of the sera.

To determine the accuracy of the ddPCR method for HBV cccDNA quantification, the plasmid containing the HBV genotype C2/Ce DNA genome (AB246345) was serially diluted from 10^5^ to 10^0^ copies/assay and determined by ddPCR and qPCR (Fig. 2A). As a result, both cccDNA-specific ddPCR and qPCR showed an excellent linear regression between expected and observed HBV cccDNA copies/assay (R^2^ = 0.9999 and R^2^ = 0.9996, respectively). The cccDNA-specific ddPCR and qPCR showed excellent linear ranges, where CV% < 20% represents complete accuracy from 10^1^ to 10^5^ copies/assay and from 10^1^ to 10^5^ copies/assay, respectively. As shown in Table S1, the cccDNA-specific ddPCR indicated that more precise than the cccDNA-specific qPCR at three concentrations (high, middle, low) using a total DNA extracted from the liver of tumor tissues in HCC patient (#4 in Table 1) for inter- and intra-assay precision.

**Table 1:** Specific detection of HBV-DNA, cccDNA, HBsAg and HBcrAg in the sera and livers of 6 selected patients.

ID	Age	Sex	HBV DNA (log copies/mL)	Anti-HBc	HBsAg (IU/mL)	HBeAg/anti-HBe	HBcrAg (LogU/mL)	Intrahepatic cccDNA (copies/1000 ng)		Intrahepatic RPP30 (copies/1000 ng)		Intrahepatic cccDNA (copies/cell)	
								Non-tumor	Tumor	Non-tumor	Tumor	Non-tumor	Tumor
1	60	M	–	+	–	−/−	< 3	n.d	n.d	82,450	51,650	–	–
2	71	M	–	+	9.2	−/+	< 3	n.d	5.0	36,100	79,400	–	0.00013
3	59	M	4.1	+	> 2000	−/+	6.76–8.74	1095	37,280	43,100	63,200	0.05081	1.17975
4	55	M	< 2.1	+	814.1	−/+	4.6	511	48,590	109,500	104,000	0.00933	0.93442
5	70	M	–	+	–	−/−	< 3	n.d	n.d	62,800	60,500	–	–
6	78	M	–	−	–	−/−	< 3	–	3.0	42,600	70,500	–	0.00009

To compare the performance of cccDNA detection between ddPCR and qPCR, we serially diluted total DNA extracted from the livers of HBV-infected chimeric mice from 20 to 0.156 ng/assay and conducted ddPCR and qPCR. As shown in Fig. 2B, the results of both ddPCR and qPCR displayed high goodness of fit (R^2^ = 0.9997 and R^2^ = 0.9685, respectively). In addition, the results of ddPCR and qPCR exhibited a near-perfect regression (R^2^ = 0.9958, p = 2.12 × 10^−70^) (Fig. 2C). These results suggest that both ddPCR and qPCR are highly sensitive and sufficiently accurate to detect twofold differences of the intrahepatic cccDNA. In contrast, when the samples were diluted to various concentrations, ranging between 5 and 8 ng/assay, ddPCR but not qPCR was able to accurately measure the differences (R^2^ = 0.9416 and R^2^ = 0.8963, respectively) (Fig. 2D). In addition, ddPCR was found to more accurately detect less than twofold difference than qPCR in other assay ranges (R^2^ = 0.9980 and R^2^ = 0.9972, respectively at higher assay range; 12–20 ng/assay and R^2^ = 0.9974 and R^2^ = 0.9946, respectively at lower assay range; 4–6 ng/assay). These results support that ddPCR could accurately detect less than twofold differences than qPCR in various range we measured. The limits of quantitative value (LOQ) and detection value (LOD) for HBV-cccDNA-specific ddPCR were determined using serial dilutions of total DNA extracted from the livers of HBV-infected and non-infected chimeric mice (Fig. 2E). The LOD and LOQ in mouse liver were 1 copy/20 ng ddPCR assay and 20 copies/20 ng ddPCR assay, respectively, and those were 5.9 copies/20 ng qPCR assay and 60.2 copies/20 ng qPCR assay, respectively. The performance of cccDNA detection between ddPCR and qPCR was also validated using the total DNA extracted from the tumor tissue of HBV-infected HCC patient #4 (Supplementary Fig. S2). The LOD and LOQ in the human liver were 1 copy/20 ng ddPCR assay and 15.8 copies/20 ng ddPCR assay, respectively, and those were 1 copy/20 ng qPCR assay and 21.6 copies/20 ng qPCR assay, respectively. Remarkably, the levels of cccDNA in the liver of the mice and humans were more sensitively detectable from a lower amount of total DNA by ddPCR compared to qPCR (Fig. 2E; Supplementary Fig. S2C).

### Specific detection of cccDNA by ddPCR

The specificity of cccDNA detection was compared between ddPCR and qPCR. Serially diluted total DNA extracted from the sera of HBV-infected chimeric mice, from 0.32 to 0.04 µl/assay, was analyzed by ddPCR and qPCR. Several studies reported that total DNA in the sera and the culture medium could contain a large amount of HBV DNA and a small amount of cccDNA^19–21^, indicating that the cccDNA-specific ddPCR of the serum should generate a very low signal. As references, the serum HBV DNA levels of the HBV-infected chimeric mice were measured using a primer–probe set that could detect both cccDNA and rcDNA (herein referred to “set 1”)^12^. As shown in Fig. S3C, the results of both ddPCR and qPCR using the set 1 primer–probe set showed high concordance (R^2^ = 0.9994 and R^2^ = 0.9906, respectively). When these serum samples were analyzed by the cccDNA specific primer–probe set without PSAD treatment, both ddPCR and qPCR was able to accurately measure cccDNA at a concentration of approximately more than 1/600 of total DNA in all dilution series, ranging between 0.32 and 0.04 µl/assay (R^2^ = 0.9022 and R^2^ = 0.9601, respectively). Moreover, when the serial diluted serum samples were measured by the cccDNA specific primer–probe set with PSAD treatment, ddPCR was able to accurately measure cccDNA at a concentration of approximately 1/1000 of total DNA in all dilution series, ranging between 0.32 and 0.04 µl/assay (R^2^ = 0.9915), but qPCR was unstable at low cccDNA concentrations (R^2^ = 0.9259, Supplementary Fig. 3C). Because expressing uPA in the liver of the uPA/SCID mice with human hepatocytes induces a sufficiently damaged liver (Tateno et al., PLoS One, 2015), we suggest that the cccDNA in hepatocytes could flow out to the blood and the cccDNA could be detectable in sera of the chimeric mice. Unfortunately, although we performed Southern blotting (SB) experiments, the mice serum volumes are too small to detect any signals after PSAD treatment; we were unable to provide data whether the detected signal was cccDNA or rcDNA in false-positive by ddPCR.

Additionally, to determine the specificity of our method for cccDNA detection in a complex background consisting of genomic DNA and rcDNA, we serially diluted HBV plasmid as templates from 10^0^ to 10^5^ copies/assay with the sera containing a large amount of HBV DNA (5.1 log IU/mL), that were obtained from inactive HBV carrier with normal liver function (#9 in Supplementary Fig. S3B). As shown in Supplementary Figure S3A, the observed cccDNA values were almost consistent with the expected cccDNA values by ddPCR (R^2^ = 0.9995). In additional experiments, cccDNA signals were not detectable in negative controls without the plasmids with and without the sera, and cccDNA values were similar between serially diluted samples with and without the sera in Fig. 2F and Supplementary Figure S3A. These data suggest that cccDNA-specific ddPCR assay could detect intrahepatic cccDNA with high specificity. Moreover, we also determined the copy number of cccDNA with PSAD digestion in 4 acute on chronic hepatitis B patients with impaired liver function (serum HBV-DNA 6.7–8.2 log IU/mL) (#1–4) and 7 inactive HB carriers with normal liver function (serum HBV DNA 5.1 log IU/mL) (#5–11) by ddPCR (Supplementary Fig. S3B). As a result, the cccDNA signals were detectable with a probability of 50% (6/12) in 4 acute on chronic hepatitis B patients with impaired liver function, while the cccDNA signal was detectable with a probability of 0% (0/21) in 7 inactive HB carriers with normal liver function. These data suggest that the cccDNA in hepatocytes might flow out to the blood and the cccDNA could be detectable in sera of the chimeric mice and humans (Supplementary Fig. S3B,C).

Finally, we compared ddPCR and SB analysis using DNA isolated from two chimeric mice in the chronic phase of HBV infection with or without ETV treatment (Supplementary Fig. S4). As shown in the Supplementary Information, these values were closely correlated with the cccDNA content detected by the SB assay.

Collectively, these results suggest that ddPCR could detect intrahepatic cccDNA with higher specificity than qPCR, especially after PSAD treatment.

### The intrahepatic HBV cccDNA level correlates strongly with serum HBV parameters

**Figure 3:**
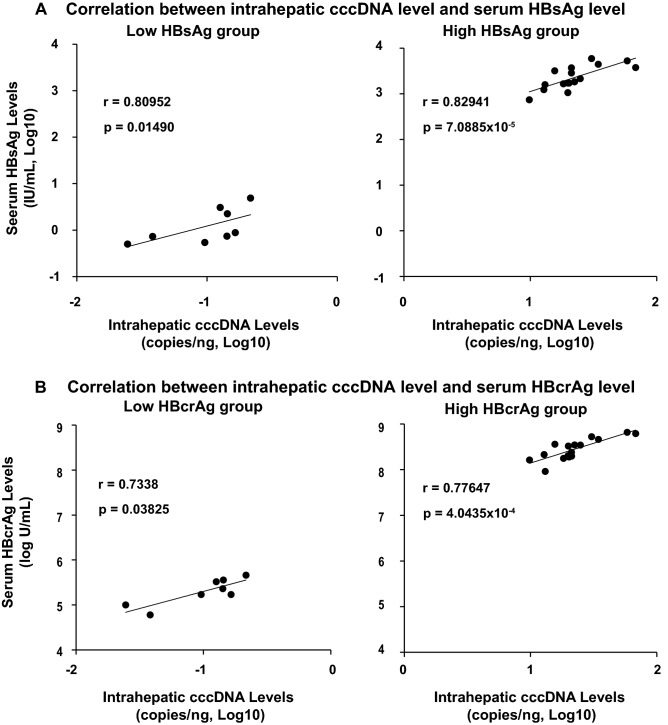
Correlation between intrahepatic cccDNA and serum HBV markers. Twenty-four HBV-infected chimeric mice under various conditions were divided into the HBV infection as during spreading phase of partially infection (n = 8; #1–2, #13–16, #19, #20) and the stable phase of fully infection (n = 16; #3–12, #17–18, #22–24). The cccDNA contents were correlated with (**A**) serum HBsAg and (**B**) serum HBcrAg in each group. Characteristics of each mouse were described in Table S2.

To determine the extent to which the intrahepatic cccDNA level is reflected by the HBV markers in the serum, the cccDNA contents were measured in 24 HBV-infected chimeric mice during 2 conditions of infection; spreading phase/partially infected mice (n = 8, low HBV group) and stable phase/fully infected mice (n = 16, high HBV group), and then correlated with the serum HBsAg and HBcrAg levels in each condition. As shown in Fig. 3A, B, the intrahepatic cccDNA levels were more significantly correlated with both the serum HBsAg level (r = 0.82941, p = 7.0885 × 10^−5^) and the HBcrAg level (r = 0.77647, p = 4.0435 × 10^−4^) in HBV high group compared with the low HBV group (r = 0.80952, p = 0.01490 and r = 0.7338, p = 0.03825, respectively), due to the small number of mice in Low HBV levels group. These results suggest that both HBcrAg and HBsAg are excellent surrogate serum markers of intrahepatic cccDNA.

### Hepatocyte proliferation has little impact on the total amount of cccDNA in the liver

**Figure 4:**
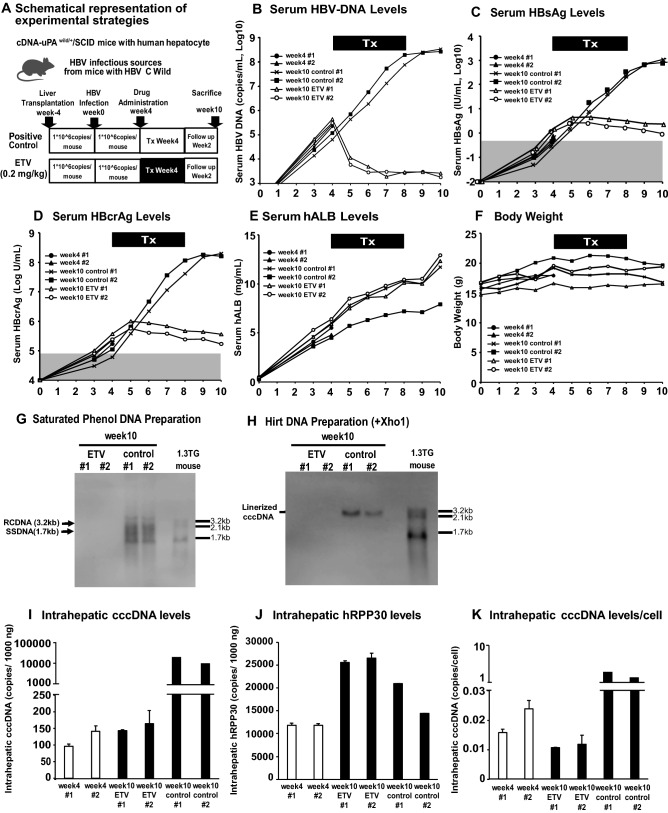
Kinetics of intrahepatic cccDNA in the chimeric mice during ETV treatment. (**A**) Schematic representation of the experiment. All six chimeric mice undergoing hepatocyte repopulation were inoculated with HBV. The two mice were treated with ETV (0.2 mg/kg/day) for 4 weeks and then followed for another 2 weeks after ETV withdrawal. The other two mice were treated with saline to serve as controls. (**B**) The serum HBV-DNA levels, (**C**) serum HBsAg levels, (**D**) serum HBcrAg levels, (**E**) human serum albumin and (**F**) their body weights were monitored during and after ETV treatment. Southern blot analysis of (**G**) HBV-DNA (rcDNA and ssDNA) and (**H**) cccDNA in the livers of HBV-infected chimeric mice, with or without ETV treatment, on week 10. The images of full length (**G** and **H**) were provided in Fig. S5 (A and B). These serum data (**B**, **C** and **D**) were associated with the levels of HBV-DNA replicative intermediates (rcDNA and ssDNA) and cccDNA in the livers of the ETV-treated and control groups by SB. (**I**) The intrahepatic cccDNA level of each chimeric mouse was determined by ddPCR. (**J**) Intrahepatic hRPP30 levels were determined by ddPCR. (**K**) Intrahepatic cccDNA levels/cell were calculated for each mouse. Values and error bars represent the means and standard deviations of two or more independent experiments. The images of the full-length membrane, with the edge of the membrane visible was provided in the original images of the supplemental information file.

Having established a highly quantitative cccDNA-specific ddPCR assay, we examined the impact of hepatocyte proliferation on the stability of cccDNA. All six chimeric mice undergoing hepatocyte repopulation were inoculated with HBV (1 × 10^6^ copies per mouse) 4 weeks after transplantation, at which time the reconstitution rate was less than 30%, based on serum human albumin (hALB) levels (Fig. 4A)^22^. As shown in Table S2, the 6 mice derived from this experiment were indicated with an asterisk (week4#1: ^*^, week4#2: ^†^, week10 ETV #1: ^‡^, week10 ETV #2: ^§^, week10 control #1: ^||^, week10 control #2: ^¶^, respectively.). Serum HBV DNA levels were measured in all mice 4 weeks later, ranging between 1.7 and 6.8 × 10^5^ copies/ml (Fig. 4B). Two chimeric mice were sacrificed on week 4 and two of the remaining four were administered ETV orally at 0.2 mg/kg/day for 4 weeks. The other two mice served as controls. The HBV DNA levels had already decreased after one week of ETV treatment and continued to decrease during the treatment, reaching a nadir on week 10 (1.6 log_10_ copies reduction) (Fig. 4B). In the ETV-treated chimeric mice, serum HBsAg and HBcrAg levels peaked at week 6 and week 5, respectively, and then decreased slightly (Fig. 4C, D). These markers continued to increase in the control group during the same period (Fig. 4B–D). Serum human albumin levels increased in both groups (Fig. 4E), reflecting liver repopulation, but the body weights did not change (Fig. 4F). As shown in Fig. 4G, H, HBV rcDNA, ssDNA and cccDNA were readily detectable in the control mice at week 10, but not in the ETV treated mice, indicating the low sensitivity of SB. The images of full length in Fig. 4G, H were provided in Fig. S5A ,B. We then analyzed the levels of intrahepatic cccDNA at weeks 4 and 10 after HBV infection (i.e. immediately before the ETV treatment (week 4) and two weeks after the termination of ETV treatment (week 10)) (Fig. 4I). Before ETV treatment, 96.0–142.0 copies of cccDNA were detected in 1000 ng of total liver DNA. Interestingly, almost the same amounts of cccDNA were detected in the ETV-treated mice at week 10 after HBV infection, consistent with the serum HBcrAg and HBsAg levels. In contrast, cccDNA levels in the control mice increased markedly to 9800 and 19,800 copies/1000 ng), a greater than 100-fold increase in 6 weeks. To determine the amount of cccDNA/hepatocyte, intrahepatic hRPP30 was quantified at the corresponding time points. As shown in Fig. 4J, the hRPP30 level increased by approximately 2.6 and 2.3-fold in the ETV-treated and control mice, in 6 weeks. The results are consistent with the increase of serum hALB levels (Fig. 4E) and suggest the ongoing human hepatocyte repopulation of the chimeric mouse livers. Consequently, the cccDNA level per hepatocyte decreased from 0.016 and 0.024 copies/cell before ETV treatment to 0.011–0.012 copies/cell after ETV treatment (Fig. 4K), while it increased to 1.361 and 1.886 copies/cell in the control mice. Collectively, these results suggest that in the liver of the 2 ETV-treated mice, the total amount of cccDNA is not affected by hepatocyte repopulation, although the cccDNA level/hepatocyte appears to be reduced after ETV treatment.

### Intrahepatic HBV cccDNA in patients with HBV-associated hepatocellular carcinoma

To apply this ddPCR-based method to clinical samples, we measured the amount of intrahepatic cccDNA and RPP30 in tumor and non-tumor tissues from five HBV-related HCC patients using the ddPCR assay. As shown in Table 1, varying levels of intrahepatic cccDNA were detected in three HBsAg-positive patients among the five HBV-related HCC patients. In two HBsAg-positive, HBcrAg-positive patients (patients 3 and 4), the cccDNA levels were 0.00933–0.05081 copies/cell in non-tumor tissues, and 0.93442–1.17975 copies/cell in tumor tissues. In the HBsAg-positive, HBcrAg-negative patient (patient 2), cccDNA was detected weakly at 0.00013 copies/cell in tumor tissue, while it was undetectable in non-tumor tissue. In two HBV-related HCC patients whose HBV infections had resolved, cccDNA levels were below the detection limit. Intrahepatic RPP30 in all 6 HCC patients, including the negative control, were 36,100–109,500 and 51,650–104,000 copies/1000 ng in non-tumor and tumor tissues, respectively. These data showed that ddPCR could detect cccDNA in tumor and non-tumor tissues.

### The impact of PEG-IFN treatment of patients with HBeAg-negative CHB on intrahepatic cccDNA levels

**Figure 5:**
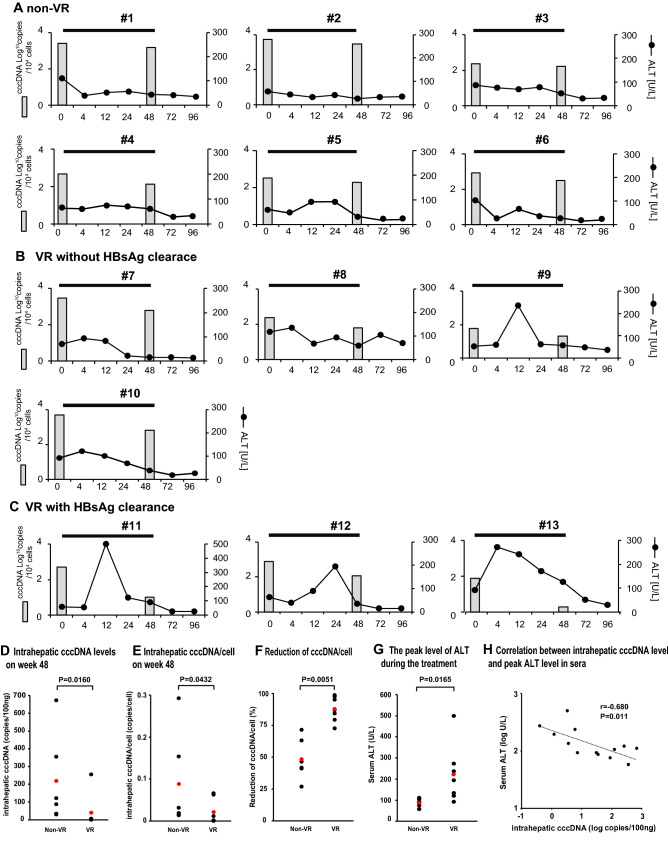
Changes of intrahepatic cccDNA content and serum ALT in CHB patients with HBV infection by PEG-IFN treatment. Effect of PEG-IFN treatment on intrahepatic cccDNA/10^4^ cells and serum ALT in CHB patients with (**A**) non-VR, (**B**) VR without HBsAg clearance and (**C**) VR with HBsAg clearance, during and after PEG-IFN treatment. These patients were treated with PEG-IFN monotherapy (180 µg/week) for 48 weeks and then followed for another 48 weeks after PEG-IFN withdrawal. (**D**) Each dot represents the average of duplicate tests for the copy number of intrahepatic cccDNA level in 100 ng total DNA of each patient. The Mann–Whitney U-test was used to examine statistical difference between the two groups. The mean values of the two groups were depicted by red dots, and were 217.7 (30.7–674.0) in the non-VR group and 39.8 (0.4–256.8) in the VR group. (**E**) Each dot represents the average of duplicate tests for the copy number of cccDNA per hepatocyte of each patient. The Mann–Whitney U-test was used to examine the statistical difference between the two groups. The mean values of the two groups were depicted by red dots, and were 0.088 (0.013–0.293) in the non-VR and 0.0206 (0.0002–0.0659) in the VR groups. (**F**) The reduction of cccDNA/cell (%) from baseline to the end of treatment was calculated in each patient, and the statistical difference was determined by the Mann–Whitney U-test. Red dots depict the mean values in the two group. (**G**) The peak levels of ALT during PEG-IFN treatment were 88.5 (58–111) in the non-VR and 222.0 (93–500) in the VR group. Comparison between two groups was performed by the Mann–Whitney U test. (**H**) Negative correlation between intrahepatic cccDNA at week 48 and the peak levels of ALT in CHB patients. The correlation between variables was analyzed using Spearman's rank correlation coefficient.

To investigate the effect of PEG-IFN on intrahepatic cccDNA, we determined the levels of intrahepatic cccDNA in patients with HBeAg-negative CHB at baseline and 48 weeks after PEG-IFN treatment (Fig. 5; Supplementary Fig. S6 and Table S3). Thirteen patients with HBeAg-negative CHB who received 48 weeks of PEG-IFN monotherapy were categorized into 3 groups: non-VR, VR without HBsAg clearance and VR with HBsAg clearance. At baseline, there was no significant difference in the levels of HBV DNA, HBsAg, and HBcrAg in the serum, or the levels of total cccDNA and cccDNA per cell in the liver, among the three groups (Supplementary Table S3). As shown in Fig. S6 and Table S3, serum HBV DNA levels in the non-VR group either did not change or decreased temporarily at the end of PEG-IFN treatment, only to rebound thereafter. In the VR without or with HBsAg clearance groups, HBV DNA levels decreased continuously after PEG-IFN treatment (Supplementary Fig. S6). Serum HBsAg levels in the non-VR and VR without HBsAg clearance groups were 2.8 ± 1.1 (1.32–3.26) and 1.1 ± 0.9 (0.40–2.38) log IU/mL, respectively, at week 48, whereas HBsAg levels were undetectable at weeks 12 and 24 in the VR with HBsAg clearance group (Supplementary Fig. S6). Serum HBcrAg levels in the non-VR group could be quantitated (4.3 ± 1.4 (2.8–6.6) Log U/mL), while those in the VR without and with HBsAg clearance groups were mostly under the quantitation limit at week 48 (Supplementary Fig. S6). Interestingly, the levels of intrahepatic cccDNA and cccDNA per cell were significantly lower in the VR groups than the non-VR group at week 48 (p = 0.0160 and p = 0.0432, respectively), associated with a significant reduction of cccDNA per cell (p = 0.0051) (all data shown in Fig. 5A–F and Supplementary Table S3). These significant changes in cccDNA levels in the VR groups were associated with a higher peak of ALT than the non-VR group (p = 0.0165) (Fig. 5A–G). As expected, the cccDNA levels at week 48 after PEG-IFN treatment significantly negatively correlated to a high peak of ALT in the patients with HBeAg-negative CHB (r = -0.680, p = 0.011) (Fig. 5H). In particular, in the VR with HBsAg clearance group, intrahepatic cccDNA levels at week 48 after PEG-IFN treatment were lower than those in the VR without HBsAg clearance group (p = 0.036), in association with the higher ALT peak (p = 0.0602) (Fig. S7A–B), although HBcrAg levels in most VR patients were undetectable, due to the limited sensitivity. Remarkably, cccDNA levels could be readily quantitated even in patients (11 and 12) with HBsAg clearance after PEG-IFN treatment (Fig. 5C; Supplementary Table S3).

## Discussion

In this study, we developed a ddPCR assay for the quantitation of HBV cccDNA, with more sensitivity, specificity and accuracy than qPCR (Figs. 1, 2; Supplementary Fig. S2, S3). The specificity of ddPCR for cccDNA was validated by the assays using the plasmid containing HBV genome and the SB assay (Fig. 2F; Supplementary Fig. S4). The intrahepatic cccDNA was closely correlated with both HBsAg and HBcrAg in the sera of HBV-infected chimeric mice (Fig. 3). Notably, the total amount of cccDNA in the repopulating liver did not change in the absence of active HBV DNA replication during ETV treatment, although the cccDNA content/hepatocyte was reduced by hepatocyte proliferation (Fig. 4). To our knowledge, this is the first study indicating the stability of cccDNA in the proliferating liver, and this novel finding could be obtained because of the high accuracy of ddPCR. The ddPCR was also able to quantitate cccDNA in CHB patients with HCC and those with HBsAg clearance after PEG-IFN treatment (Table 1 and Table S3). Importantly, ddPCR readily quantitated the intrahepatic cccDNA content, even after HBsAg clearance (Fig. 5).

It is generally believed that the amount of cccDNA should be reduced to a very low level to cure HBV infection. Thus, a reliable assay to measure cccDNA is required to evaluate the therapeutic efficacy of new anti-HBV treatments. While qPCR measures the fluorescence signal to determine the amount of DNA in the sample, ddPCR measures the positive and negative fluorescence data from a sample droplet and fits it to a Poisson distribution to measure the endpoint. Therefore, ddPCR reduces the influence of PCR inhibitors, which affect amplification efficiency, and ddPCR is a new technique with improved quantification^4^. Furthermore, ddPCR was reported to be less variable between experiments than qPCR, allowing long-term monitoring^23^. Several recent studies have applied the ddPCR technology to measure HBV cccDNA levels in cultured cells and the livers of CHB patients, and demonstrated the higher sensitivity of ddPCR than qPCR^24–26^.

As explained above, several studies both ddPCR and qPCR technologies have already utilized to measure cccDNA using cccDNA-specific primers and probes. However, although the number of mice for this study is small, we believe this is the first study that applied the ddPCR technology to test the impact of hepatocyte proliferation on the stability of cccDNA. We found that the cccDNA content in the liver is extremely stable during 2–3 times of cell division, and this discovery was possible because of the highly sensitive methods such as ddPCR. Previous study has already used the ddPCR method to study the effect of nucleoside analogue on intrahepatic cccDNA levels but did not address the issue of IFN-treated patients^24^. We subsequently showed that our ddPCR can be applied to human samples including HCC and IFN-treated patients.

Although we could not distinguish whether the signals detected in serum of the chimeric mice were false positive rcDNA or cccDNA by SB, cccDNA signals were not detected in negative controls with and without the sera, and cccDNA copies were similar between with or without the sera, suggesting that the ddPCR assay could detect cccDNA with high specificity, and the assay could detect cccDNA in mice serum. In the current study, the novel ddPCR assay enabled more specific detection of small amounts of cccDNA, even in the presence of large amounts of HBV DNA, indicating more accuracy, specificity and sensitivity than qPCR (Fig. 2; Supplementary Fig. S2, S3). Our results and the previous findings demonstrated that ddPCR is more precisely than qPCR^23^ (Supplementary Table S1), ddPCR might be more appropriate for measuring cccDNA levels than qPCR in clinical settings, because most anti-HBV drugs do not directly target cccDNA and, therefore, require long-term treatment to achieve a meaningful cccDNA reduction.

The high accuracy, specificity and sensitivity of ddPCR allowed us to determine the impact of hepatocyte proliferation on cccDNA. Surprisingly, the total amount of cccDNA in the liver showed no reduction, even though the number of hepatocytes doubled in the absence of active HBV replication (Fig. 4I, week 4 vs. week 10 with ETV). Because of the small number of mice involved, however, these results are not statistically significant. Nevertheless, the data suggest that cccDNA is extremely stable during hepatocyte repopulation. Our findings differ from those recently reported by Allweiss et al.; total cccDNA levels in the liver appeared to be reduced by the proliferation of hepatocytes^27^. This discrepancy presumably reflects different experimental conditions. In their study, HBV-infected hepatocytes were transplanted to uPA-SCID mice, and the baseline cccDNA levels were measured almost immediately after the transplantation of HBV-infected hepatocytes under conditions leading 1000 times hepatocyte division. In contrast, as HBV could efficiently infect with the chimeric mice with a replacement rate of more than 30% of human hepatocytes in our PXB-mice (personal communication), we inoculated HBV at 4 weeks after liver transplantation and conducted the NA administration test with reference to human ALB value that predicts the replacement rate of human hepatocytes. Hence, our experiments would have only 2–3 times hepatocyte divisions, the stability of cccDNA during multiple hepatocyte divisions needs to be investigated in the future. This notion is in line with the observations in NA treated woodchucks chronically infected with woodchuck hepatitis virus^28^ and growing ducklings infected with duck HBV^29^. These observations support the hypothesis that some fraction of cccDNA was distributed to the daughter cells of infected hepatocytes (Supplementary Fig. S9A). Alternatively, it is possible that the liver reconstitution after HBV infection mainly reflected the proliferation of uninfected hepatocytes, while the majority of HBV-infected hepatocytes did not proliferate during the same period of time (Supplementary Fig. S9B). Because of the small number of mice involved in this study, we did not conclude the significance of cccDNA maintenance in cell division, and further studies are required to distinguish these alternatives.

Secreted HBcrAg is considered a good surrogate marker for intrahepatic cccDNA. Also, middle and large HBsAg might reflect the activity of cccDNA, although HBsAg is partially driven from integrated HBV DNA^30^. Patients with low serum HBsAg and HBcrAg levels have been reported to have a low risk of relapse after cessation of NAs therapy^6,8,31–33^. Recent studies indicated that serum HBcrAg was a better surrogate marker of intrahepatic cccDNA than serum HBsAg^34–36^. Quantitation of cccDNA by ddPCR recapitulated those previous findings, as serum HBcrAg was also correlated with intrahepatic cccDNA more significantly than HBsAg, in HBeAg-negative CHB patients (p = 8.4624 × 10^−10^ and p = 0.000628, respectively, Supplementary Fig. S8). In human samples, HBcrAg was more closely correlated with cccDNA than HBsAg. Besides, a good positive correlation between serum HBsAg and cccDNA was obtained in chimeric mice with immunodeficiency (Fig. 3). This discrepancy presumably reflects the presence of anti-HBs and/or the presence of HBsAg-producing viral integrations in human samples. As expected, the more profound reduction of cccDNA in the VR than non-VR groups was associated with higher peak ALT activities after PEG-IFN treatment (Fig. 5), indicating a role of cell death in elimination of cccDNA. Interestingly, Lucifora et al. suggested that APOBEC3 family cytidine deaminase A3A mediates the IFN-a induced cccDNA degradation^37^.

In conclusion, a sensitive, specific, highly quantitative ddPCR was developed for the measurement of cccDNA in the liver. This new method revealed that intrahepatic cccDNA is stable during hepatocyte proliferation and persists at quantifiable levels, even after serum HBsAg clearance. The use of ddPCR will provide valuable information on the development of new drugs against CHB infection.

## Supplementary Information

Supplementary Information.

## Abbreviations

cccDNA: Covalently closed circular DNA
CHB: Chronic hepatitis B
ddPCR: Droplet digital PCR
SB: Southern blotting
HBsAg: Hepatitis B surface antigen
HBcrAg: Hepatitis B core-related antigen
HCC: Hepatocellular carcinoma
PEG-IFN: Pegylated interferon alpha-2a
VR: Virological response
HBV: Hepatitis B virus
NAs: Nucleos(t)ide analogs
ETV: Entecavir
qPCR: Quantitative real-time PCR
uPA^wild^^/^^+^/SCID + / + mice: Severe combined immunodeficiency mice transgenic for the urokinase-type plasminogen activator gene
Human-liver-chimeric mice: The liver replaced with human hepatocytes
HBeAg: Hepatitis B e antigen
PSAD: Plasmid-safe ATP dependent DNase
hALB: Human albumin
rcDNA: Relaxed circular DNA
DRs: Direct repeats
nt: Nucleotides
