# Supplementary material for: Droplet digital PCR assay provides intrahepatic HBV cccDNA quantification tool for clinical application
Source: Sci Rep. 2022 Feb 8;12:2133. doi: 10.1038/s41598-022-05882-9 (PMC8826402; doi:10.1038/s41598-022-05882-9)
Supplement: Supplementary file 1 — Supplementary Information. [file 41598_2022_5882_MOESM1_ESM.pdf]

Supplementary Information

Droplet digital PCR assay provides intrahepatic HBV cccDNA quantification tool for clinical application

Sanae Hayashi[1,7], Masanori Isogawa[1], Keigo Kawashima[1], Kyoko Ito[1], Natthaya Chuaypen[2], Yuji Morine[3], Mitsuo Shimada[3], Nobuyo Higashi-Kuwata[4], Takehisa Watanabe[7], Pisit Tangkijvanich[2], Hiroaki Mitsuya[4,5,6], Yasuhito Tanaka[1,7*]

[1]Department of Virology & Liver Unit, Nagoya City University Graduate School of Medical Sciences, Nagoya, Japan; [2]Center of Excellence in Hepatitis and Liver Cancer, Faculty of Medicine, Chulalongkorn University, Bangkok, Thailand; [3]Department of Surgery, Tokushima University, Tokushima, Japan; [4]Department of Refractory Viral Infections, National Center for Global Health & Medicine Research Institute, Tokyo, Japan; [5]Experimental Retrovirology Section, HIV and AIDS Malignancy Branch, National Cancer Institute, National Institutes of Health, Bethesda, MD, USA; [6]Department of Clinical Sciences, Kumamoto University Hospital, Kumamoto, Japan; [7]Department of Gastroenterology and Hepatology, Faculty of Life Sciences, Kumamoto University, Kumamoto, Japan.

Materials and Methods

Chimeric mice with human hepatocytes.

Severe combined immunodeficiency mice transgenic for the urokinase-type plasminogen activator gene (cDNA-uPA$^{wild/+}$/SCID+/+ mice), with their livers replaced by human hepatocytes, (human-liver-chimeric mice) were purchased from PHOENIXBIO Co., Ltd. (Hiroshima, Japan). The efficiency of liver repopulation was estimated by the human albumin (hALB) level in the serum, as described previously [1]. The animal protocol was approved by the Ethics Committees of PHOENIXBIO Co., Ltd (Permit Number: 1910). These mice were infected with sera obtained from human hepatocyte chimeric mice previously infected with genotype C2/Ce, as described in a previous report [2].

Patients.

A total of 6 HCC patients and 13 hepatitis B e antigen (HBeAg)-negative patients with CHB were enrolled in this study. Liver tumor tissues and the corresponding non-tumor tissues were collected from five patients with HBV infection (3 HBsAg-positive and 2 HBsAg-negative/anti-HBc-positive) and one patient without HBV infection (HBsAg-negative and anti-HBc-negative) at Tokushima University Hospital and Nagoya City University Hospital in Japan. Thirteen HBeAg-negative patients who received 48 weeks of PEG-IFN-alfa2a (180 μg/week) monotherapy at the King Chulalongkorn Memorial Hospital, Bangkok, Thailand, were enrolled to this study, and paired liver tissues were collected before and after PEG-IFN treatment. A virological response (VR) was defined as an HBV DNA level less than 2,000 IU/mL at 48 weeks after treatment. Written informed consent was obtained from each patient and the study protocol conformed to the ethical

guidelines of the Declaration of Helsinki and was approved by the appropriate institutional ethics review committees of each institute.

Analysis of virological markers.

HBV DNA was extracted from 5 μl of mouse serum using SMITEST EX-R+D KIT (MEDICAL AND BIOLOGICAL LABORATORIES). A real-time PCR assay (Taqman) was used for DNA quantification. HBV DNA titers of the chimeric mice were measured by real-time PCR, as described previously [3] [4]. Hepatitis B surface antigen (HBsAg) and hepatitis B core-related antigen (HBcrAg) were measured by chemiluminescent enzyme immunoassays, using commercial kits (FUJIREBIO Inc., Tokyo, Japan), as described previously [2] [5]. The detection limits of the HBsAg and HBcrAg assays are 0.005 IU/ml and 3 logU/ml, respectively. The HBV DNA titers of the patients were measured using a TaqMan polymerase chain reaction assay (COBAS TaqMan, ROCHE MOLECULAR SYSTEMS [lower detection limit: 20 IU/ml]) or Abbott RealTime HBV assay (lower detection limit: 10 IU/ml).

Isolation of genomic DNA from the livers of chimeric mice.

Genomic DNA was isolated from liver tissue using the phenol/chloroform method, as described previously [6]. Briefly, 50 mg of mouse liver were lysed with 5 ml of lysis buffer containing 10 mM Tris–HCl, pH 8.0, 10 mM EDTA, 10 mM NaCl, 0.5% SDS and 500 ng of proteinase K at 37°C overnight with gentle rotation. After incubation, an equal volume of Tris-saturated phenol was added and then rotated for 30 min at room temperature. The lysate was then clarified by centrifugation at 2,500 rpm for 10 min at 4°C and the

supernatant was extracted twice with the phenol and once with chloroform: isoamyl alcohol. Approximately 0.1 volume of 3M sodium acetate and 2.5 volumes of pre-cooled ethanol were added to the DNA sample, followed by incubation at -80ºC for 1 hour and centrifugation at 3,000 rpm for 15 min at 4ºC. The precipitate was washed with 70% ethanol and resuspended in 300 µl TE buffer (10 mM Tris–HCl, pH 8.0, 1 mM EDTA).

Protein free-DNA extraction from the mouse liver was carried by a modified Hirt extraction procedure [7]. Briefly, 100 mg of mouse liver were lysed in 4.5 ml of 10 mM Tris–HCl (pH 7.5), 10 mM EDTA and 0.6% SDS. After 30 min incubation at room temperature, the lysate was transferred into a 15 ml tube followed by addition of 1.2 ml of 5 M NaCl and incubation at 4ºC overnight. The lysate was then clarified by centrifugation at 10,000 rpm for 30 min and extracted twice with Tris-saturated phenol and once with phenol: chloroform. DNA was precipitated with two volumes of ethanol overnight at room temperature. After centrifugation at 4,000 rpm at 4ºC for 30 min, the DNA was washed with 75% ethanol and resuspended in TE buffer (10 mM Tris–HCl, pH 8.0, 1 mM EDTA).

The genomic DNA and protein free-DNA isolated from the livers of chimeric mice with HBV infection was treated with DNase-free RNase and digested with a restriction enzyme (*Hind* III) before being analyzed.

Quantification of HBV cccDNA and relaxed circular (rc) DNA by ddPCR.

For HBV cccDNA quantitation of the liver DNA extracts, 1 µg of total liver DNA was treated for 30 minutes at 37°C with 10 U of plasmid-safe ATP dependent DNase (PSAD) (EPICENTRE, Madison, Wisconsin, USA) to digest single-stranded DNA and linear double-strand DNA. PSAD digestion was carried out in a 50 µl reaction containing 5 µl

10x PSAD buffer, 2 µl 25 mM ATP, 1 µl PSAD, 33 µl deionized water and 9 µl (20-1000 ng) total liver DNA. DNA samples pre-digested with PASD were used as template inputs for either qPCR or ddPCR amplification. Specific primers for cccDNA amplification (targeted across the single-stranded (SS) gap region of relaxed circular HBV DNA (rcDNA) were designed as shown in Fig. 1A, B. The sequences of the forward and reverse primers were 5'-ACGGGGCGCACCTCTCTTTACGCGG-3' [nt: 1519-1543] and 5'-CAAGGCACAGCTTGGAGGCTTGAAC-3' [nt: 1862-1886], respectively, and the probe 5'-FAM-AACGACCGACCTTGAGGCAT-MGB-3'. A 20 µl ddPCR reaction mix comprised 10 µl 2x ddPCR Supermix for probes (no dUTP) (BIO-RAD LABORATORIES, Pleasanton, California, USA), 0.86 µl 100 µM primers and 10 µM probe mix (900 nM and 250 nM), 1 µl 5 U restriction enzyme, 7.14 µl deionized water and 1 µl digested/purified DNA sample. Among the restriction enzymes recommended by the manufacturer (i.e., Alul, CviQ1, Dpn II, Hae III, Mse I, and Hind III), only Hae III was found not to digest the targeted region for cccDNA detection. There are fifteen restriction sites for Hae III in the HBV genome outside the cccDNA amplification region. Reaction droplets were generated according to the manufacturer's protocol using a QX200 Droplet Generator (BIO-RAD LABORATORIES, Hercules, California, USA). Intrahepatic HBV cccDNA was amplified using a C1000 touch Thermal Cycler (BIO-RAD LABORATORIES, Hercules, California, USA) with the following amplification profile: an enzyme activation step of 10 min at 95°C, followed by 40 cycles of denaturation for 30 s at 94°C, annealing/extension for 1 min at 61.2°C and an enzyme deactivation of 10 min at 98°C. After amplification, positive and negative droplets were quantified by a QX200 Droplet Reader (BIO-RAD LABORATORIES, Hercules,

California, USA) using QuantaSoft software version 1.7 (BIO-RAD LABORATORIES, Hercules, California, USA). All intrahepatic HBV cccDNA values were normalized to cell number assessed by hRPP30 copy number variation assay (BIO-RAD LABORATORIES, Pleasanton, California, USA) [8].

Quantification of HBV cccDNA by qPCR.

Real-time PCR was performed in a StepOnePlus™ Real-Time PCR System (THERMO FISHER SCIENTIFIC, Waltham, MA) using a 25 µl reaction volume containing 100 ng DNA (for cccDNA quantification, a volume equivalent to 100 ng before PSAD treatment), 12.5 µl TaqMan™ Gene Expression Master Mix, 0.3 µmol/l forward and reverse primers, and 0.1 µmol/l probe. Amplification of cccDNA was performed as follows: 95°C for 10 minutes then 50 cycles of 95°C for 15 seconds and 65°C for 60 seconds. The resultant Ct values were analyzed by StepOne and StepOnePlus Software v2.3 (THERMO FISHER SCIENTIFIC, Waltham, MA). Serial dilutions of a plasmid containing an HBV monomer (pHBV: AB246337) served as quantification standards.

Specific detection of cccDNA by ddPCR.

ddPCR and Southern blot (SB) analysis were compared using DNA isolated from two chimeric mice (mouse #1 without ETV and mouse #2 with ETV) in the chronic phase of HBV infection (Supplementary Fig. S4). As shown in Fig. S4A, the amounts of cccDNA detected by ddPCR were 1185.5 and 1428.5 copies/20 ng. SB assay loaded side-by-side on the same gel for direct comparison of both non-linearized and linearized Hirt extracted DNA (mouse #1 without ETV and mouse #2-4 with ETV). The non-linearized Hirt

extracted cccDNA and protein-free rcDNA (Xho1-) were observed around 2.1 kb and 3.5 kb, respectively. The linearized Hirt extracted DNA with Xho1 (Xho1+), both bands around 2.1 kb and 3.5 kb migrated together at 3.2 kb. Importantly, the amounts of cccDNA detected by ddPCR were well-correlated with the cccDNA content (Xho1-) detected by the SB assay (Supplementary Fig. S4A and B; mouse #1 without ETV and mouse #2 with ETV). The copy numbers of cccDNA per hepatocyte were examined by correcting the cccDNA content by the amount of a single copy gene, hRPP30 (Supplementary Fig. S4C, D, and E). Because only human hepatocytes harbor hRPP30 in these human hepatocyte chimeric mice, cccDNA/hepatocyte could be calculated by dividing the amount of cccDNA by hRPP30 divided by 2 (Supplementary Fig. S4E). The intrahepatic cccDNA levels in the two HBV infected chimeric mice were 446.7 and 398.0 copies/20 ng, while the levels of hRPP30 in the corresponding mice were 346.7 and 335.3 copies/20 ng, indicating that, on average, one hepatocyte in these mice harbored approximately 2.37-2.57 copies of cccDNA. In contrast, cccDNA was barely detectable in non-infected mice (0.9 copies/20 ng) (Supplementary Fig. S4C), although the level of hRPP30 was similar (499.3 copies/20 ng) to that in the HBV-infected mice.

References

[1] Tateno, C. *et al*. Near completely humanized liver in mice shows human-type metabolic responses to drugs. *Am J Pathol* **165**, 901-912 (2004)

[2] Sugiyama, M. *et al*. Influence of hepatitis B virus genotypes on the intra- and extracellular expression of viral DNA and antigens. *Hepatology.* **44**, 915-924 (2006).

[3] Tanaka, Y. *et al*. A case-control study for differences among hepatitis B virus infections of genotypes A (subtypes Aa and Ae) and D. *Hepatology.* **40**, 747-755 (2004).

[4] Abe, A. *et al.* Quantitation of hepatitis B virus genomic DNA by real-time detection PCR. *Journal of clinical microbiology.* **37**, 2899-2903 (1999).

[5] Kimura, T. *et al*. Hepatitis B virus DNA-negative dane particles lack core protein but contain a 22-kDa precore protein without C-terminal arginine-rich domain. *The Journal of biological chemistry.* **280**, 21713-21719 (2005).

[6] Mayer MP. A new set of useful cloning and expression vectors derived from pBlueScript. *Gene.* **163**, 41-46 (1995).

[7] Zhou, T. *et al*. Hepatitis B virus e antigen production is dependent upon covalently closed circular (ccc) DNA in HepAD38 cell cultures and may serve as a cccDNA surrogate in antiviral screening assays. *Antiviral Res.* **72**, 116-124 (2006).

[8] Dyavar, SR. *et al.* Normalization of cell associated antiretroviral drug concentrations with a novel RPP30 droplet digital PCR assay. *Sci Rep.* **8**, 3626 (2018).

Table S1. An Intra-assay and inter-assay precision of ddPCR-cccDNA assay using human sample

	ddPCR			qPCR		
samples	cccDNA levels (mean copies/assay)	SD	CV(%)	cccDNA levels (mean copies/assay)	SD	CV(%)
Intra-assay Precision						
high	147.2	17.0	11.6	80.21	8.6	10.7
middle	41.6	4.3	10.3	20.37	3.4	16.7
low	18.52	2.9	15.6	10.31	2.1	20.3
Inter-assay Precision						
high	35.4	3.0	8.5	22.6	4.3	18.9
middle	18.3	0.7	4.0	10.7	1.8	16.6
low	8.4	0.6	7.5	5.4	1.3	23.5

Table S2. Specific detection of HBV DNA, cccDNA, HBsAg and HBcrAg in sera and livers of 24 selected mice.

Mouse ID	After inoculation (days)	HBV DNA (copies/mL)	cccDNA (copies/ng)	cccDNA (copies/cell)	HBsAg (IU/mL)	HBcrAg (Log U/mL)	Time of NAs after inoculation (days)	Hepatocyte phase at the time of inoculation
1[*]	28	$2.1\ x10^5$	0.10	0.016	0.54	5.2	none	Repopulation
2[†]	28	$2.6\ x10^5$	0.14	0.024	0.74	5.4	none	Repopulation
3[‡]	71	$2.6x10^8$	9.8	1.361	737.67	8.2	none	Repopulation
4[§]	71	$3.2x10^8$	19.8	1.886	1058.39	8.3	none	Repopulation
5	99	$3.7x10^8$	13.067	1.7859	1587.88	8.0	none	Repopulation
6	99	$1.4x10^8$	12.80	1.8641	1228.66	8.3	none	Repopulation
7	113	$1.3x10^9$	68.0	5.35	3744.75	8.8	none	After repopulation
8	113	$1.6x10^9$	58.3	4.14	5269.83	8.8	none	After repopulation
9	127	$3.3x10^8$	22.33	2.5769	1828.84	8.5	none	Repopulation
10	127	$2.6x10^8$	19.9	2.3738	1711.41	8.5	none	Repopulation
11	137	$9.9x10^8$	34.4	3.38	4429.21	8.7	none	After repopulation
12	137	$7.6x10^8$	24.8	2.89	2141.68	8.5	none	After repopulation
13[‖]	71	$1.8x10^3$	0.165	0.012	0.88	5.2	+ (29-57)	Repopulation
14[¶]	71	$2.6x10^3$	0.143	0.011	2.25	5.6	+ (29-57)	Repopulation
15	99	$4.3x10^3$	0.02	0.0018	0.5	5.0	+ (29-85)	Repopulation
16	99	$5.2x10^3$	0.22	0.0176	4.9	5.7	+ (29-85)	Repopulation
17	109	$1.5x10^6$	30.4	3.15	5904.09	8.7	+ (67-108)	After repopulation
18	109	$3.1x10^6$	15.6	1.51	3170.18	8.6	+ (67-108)	After repopulation
19	127	$8.4x10^3$	0.038	0.0025	0.73	4.8	+ (29-113)	Repopulation
20	127	$1.9x10^4$	0.1	0.0081	3.07	5.5	+ (29-113)	Repopulation
21	137	$6.6x10^6$	20.2	1.86	1710.46	8.3	+ (67-108)	After repopulation
22	137	$4.2x10^6$	18.3	1.60	1646.94	8.2	+ (67-108)	After repopulation
23	137	$4.5x10^6$	21.1	1.60	2846.6	8.3	+ (67-108)	After repopulation
24	137	$1.0x10^7$	21.1	1.73	3690.15	8.4	+ (67-108)	After repopulation

Table S3. HBV DNA, HBsAg, HBcrAg, cccDNA, RPP30 and cccDNA/cell at baseline and at the end of PEG-IFN treatment.

				At baseline						At the end of PEG-IFN treatment					
Case	Group	Sex	Age	HBV DNA (logU/mL)	HBsAg (logIU/mL)	HBcrAg (logU/mL)	cccDNA (copies/ 100 ng)	RPP30 (copies/ 100 ng)	cccDNA/ cell	HBV DNA (logU/mL)	HBsAg (logIU/L)	HBcrAg (logU/mL)	cccDNA (copies/ 100 ng)	RPP30 (copies/ 100 ng)	cccDNA/ cell
1	non-VR	M	48	5.5	3.53	5.5	1170.0	8970	0.261	6.2	3.26	5.0	674.0	8750	0.154
2	non-VR	M	30	7.0	3.58	6.5	2038.0	7480	0.545	6.1	2.97	6.6	356.0	2430	0.293
3	non-VR	M	43	4.8	2.81	3.3	145.3	12900	0.023	1.9	1.79	2.8	35.0	4240	0.017
4	non-VR	M	31	5.2	3.35	4.7	248.0	10550	0.047	1.7	3.26	3.5	88.0	13100	0.013
5	non-VR	M	51	5.9	4.47	3.7	112.7	6880	0.033	1.5	4.25	3.6	30.7	3210	0.019
6	non-VR	F	40	4.9	3.22	4.2	76.0	1790	0.085	2.6	1.32	4.2	122.7	7790	0.031
7	VR without HBsAg clearance	M	20	7.4	3.58	5.9	2040.0	13600	0.300	1.5	2.38	3.2	7.2	230	0.063
8	VR without HBsAg clearance	M	43	6.1	3.06	4.7	160.0	13300	0.024	<1	0.86	2.0	3.6	6050	0.001
9	VR without HBsAg clearance	M	42	5.2	2.88	3.8	34.0	12200	0.006	<1	0.82	2.0	6.0	7830	0.002
10	VR without HBsAg clearance	M	36	8.3	4.20	6.5	4068.0	16200	0.502	2.9	0.40	4.4	256.8	7790	0.066
11	VR with HBsAg clearance	M	42	5.1	2.80	n.a.	432.0	16500	0.052	<1	<-1.3	n.a.	3.3	9350	0.001
12	VR with HBsAg clearance	M	49	4.8	2.84	3.5	4.1	109	0.076	<1	<-1.3	2.0	1.2	200	0.012
13	VR with HBsAg clearance	M	50	5.1	3.18	2.9	11.3	2910	0.008	<1	<-1.3	2.0	0.4	4230	0.0002

Figure S1

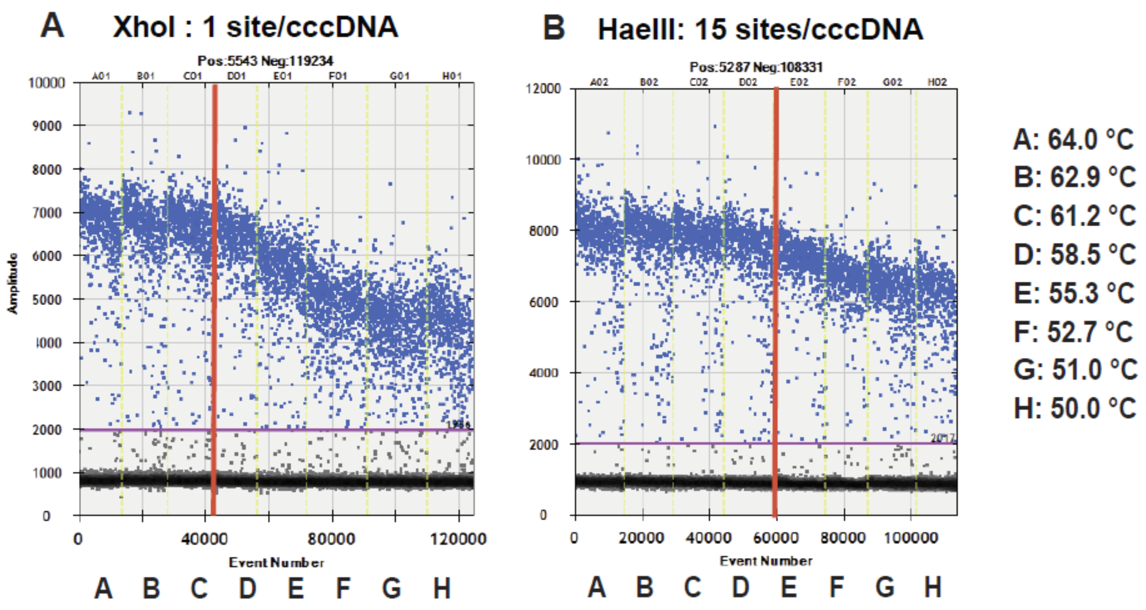

Figure S1. Adjustment of the ddPCR conditions for HBV cccDNA quantification.

Effect of restriction enzymes on the liver DNA of chimeric mice infected with HBV. Temperature gradient ddPCR for cccDNA detection using Xho I (A) or Hae III (B). Both panels show how the positive droplet populations change with decreasing annealing temperatures. The overall fluorescence of the positive droplet population (in blue) was higher at higher annealing temperatures and reduced at lower annealing temperatures.

Figure S2

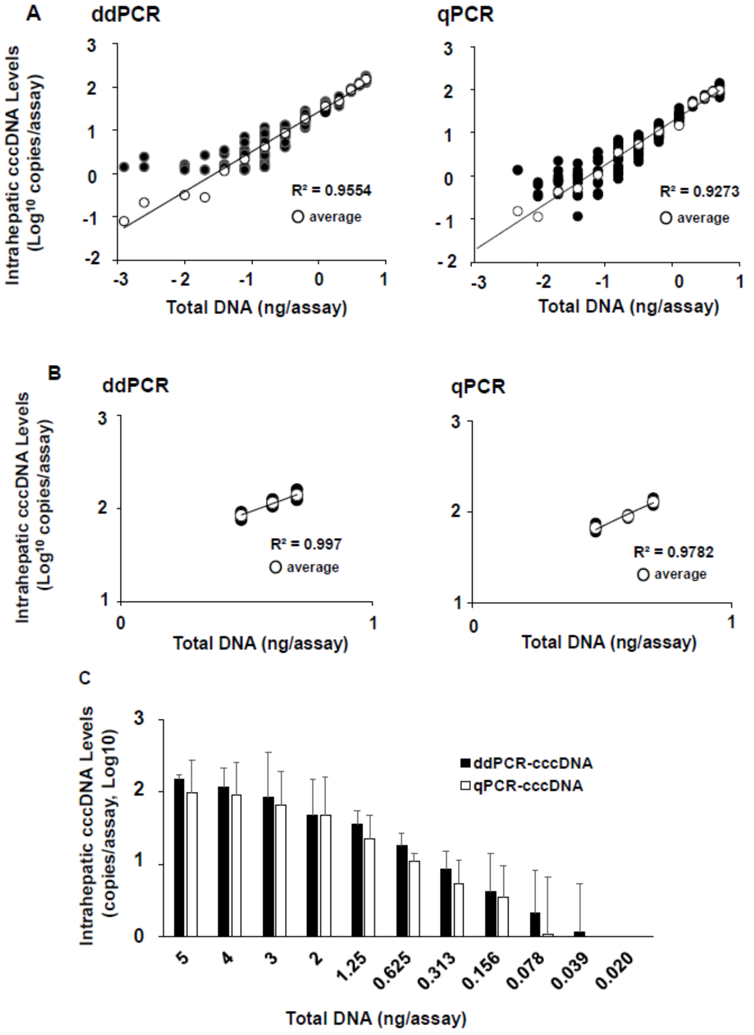

Fig. S2. Comparison of ddPCR to real-time PCR for quantitation of cccDNA.

(A) Intrahepatic cccDNA levels in the tumor tissues of patient infected with HBV infection (#4 in Table 1) were measured using various amounts of DNA ranging from 5 to 0.00125 ng/assay by ddPCR or real-time PCR. (B) Intrahepatic cccDNA levels from the tumor tissues were measured using various amounts of DNA, ranging from 3 to 5 ng/assay. (C) Intrahepatic cccDNA levels from the tumor tissues were measured using various amounts of DNA ranging from 5 to 0.00125 by ddPCR or real-time PCR. Each assay was conducted in more than triplicate. Target concentrations were repeatedly and independently tested three times.

Figure S3

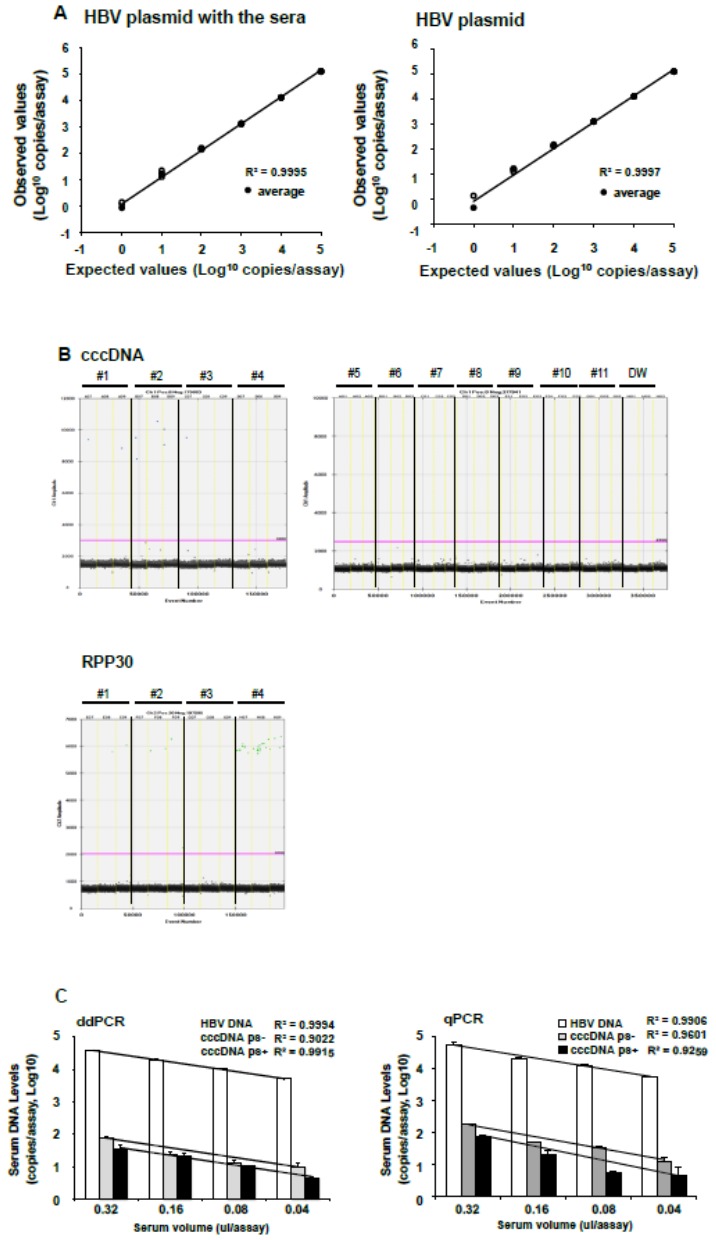

Fig. S3. The specificity of ddPCR for quantitation of cccDNA.

(A) Amplification of a plasmid containing the HBV genotype C2/Ce DNA genome (AB246345) using a 10-fold dilution of DNA ranging from 10^5 to 10^0 copies/assay with or without the sera from IC patient (#9 in Fig.S3C) by ddPCR. (B) Total DNA isolated from the

sera of 4 acute on chronic hepatitis B patients with impaired liver function (serum HBV DNA 6.7-8.2 log IU/mL) (#1-4) and 7 inactive HBV carries with normal liver function (serum HBV DNA 5.1 log IU/mL) (#5-11) was analyzed for cccDNA and RPP30. (C) Total DNA isolated from the sera of chimeric mice was analyzed for HBV DNA and cccDNA. Serum HBV DNA levels were measured using a primer-probe set that detects both cccDNA and rcDNA. Serum cccDNA levels were determined using the cccDNA-specific primers and probe set by ddPCR or real-time PCR, with PSAD treatment.

Figure S4

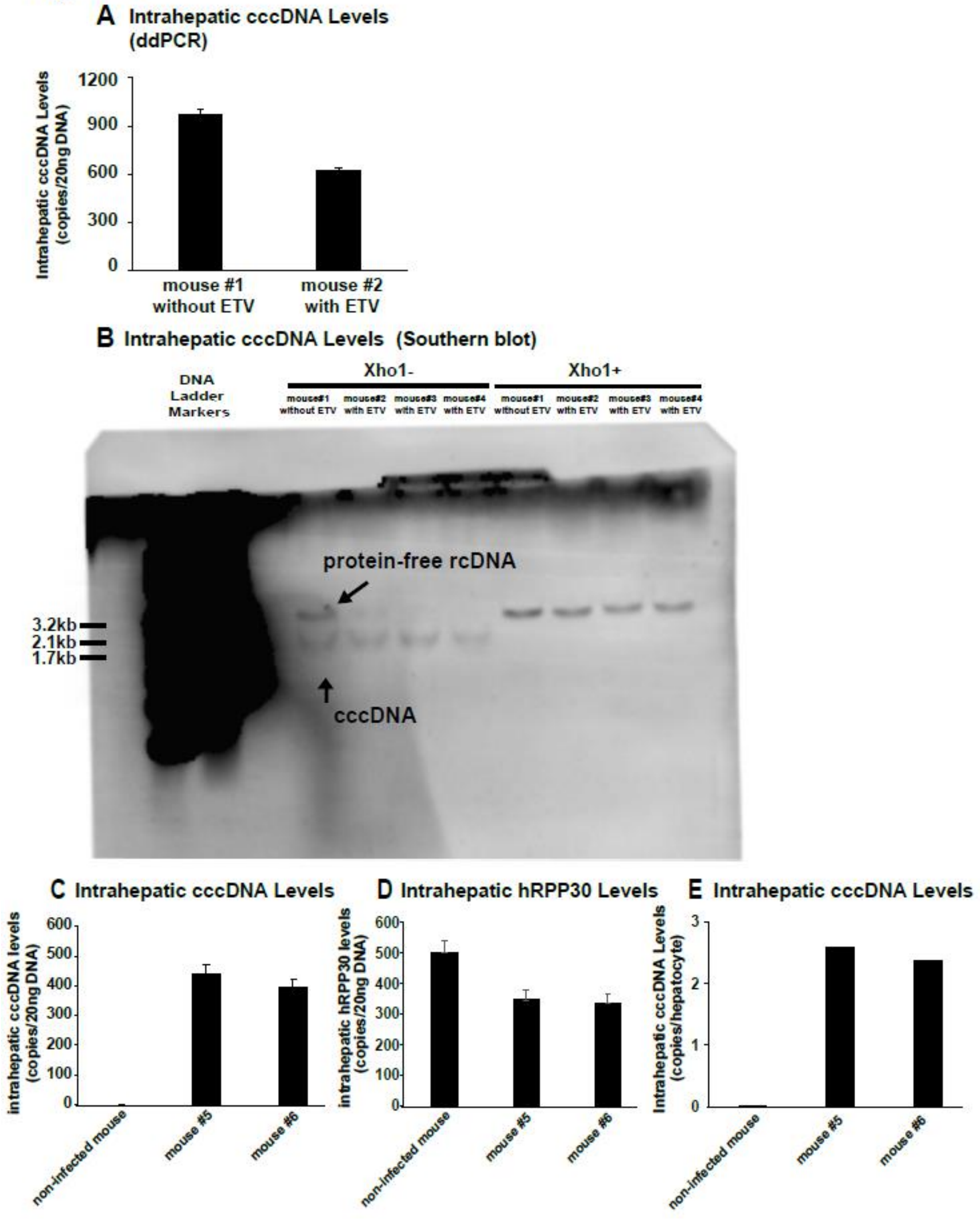

Figure S4. Comparison between ddPCR assay and Southern blot analysis for measuring intrahepatic cccDNA.

(A)Intrahepatic HBV cccDNA levels in HBV-positive mouse livers (mouse #1 without ETV and mouse #2 with ETV) were analyzed by ddPCR. (B) The intrahepatic DNA (mouse #1

without ETV and mouse #2-4 with ETV) extracted by the Hirt method was subjected to Southern blot (SB) analysis. SB assay loaded side-by-side on the same gel for direct comparison of both non-linearized and linearized Hirt extracted DNA. The non-linearized Hirt extracted cccDNA and protein-free rcDNA (Xho1-) were observed around 2.1 kb and 3.5 kb, respectively. The linearized Hirt extracted DNA with Xho1 (Xho1+), both bands around 2.1 kb and 3.5 kb migrated together at 3.2 kb. The membrane was photographed out of the proper position for imaging, so we are unable to provide the full-length gels. (C) Intrahepatic cccDNA levels and (D) hRPP30 levels in mice with or without HBV infection were analyzed by ddPCR. (E) cccDNA/hepatocyte was calculated by dividing cccDNA by hRPP30 divided by 2.

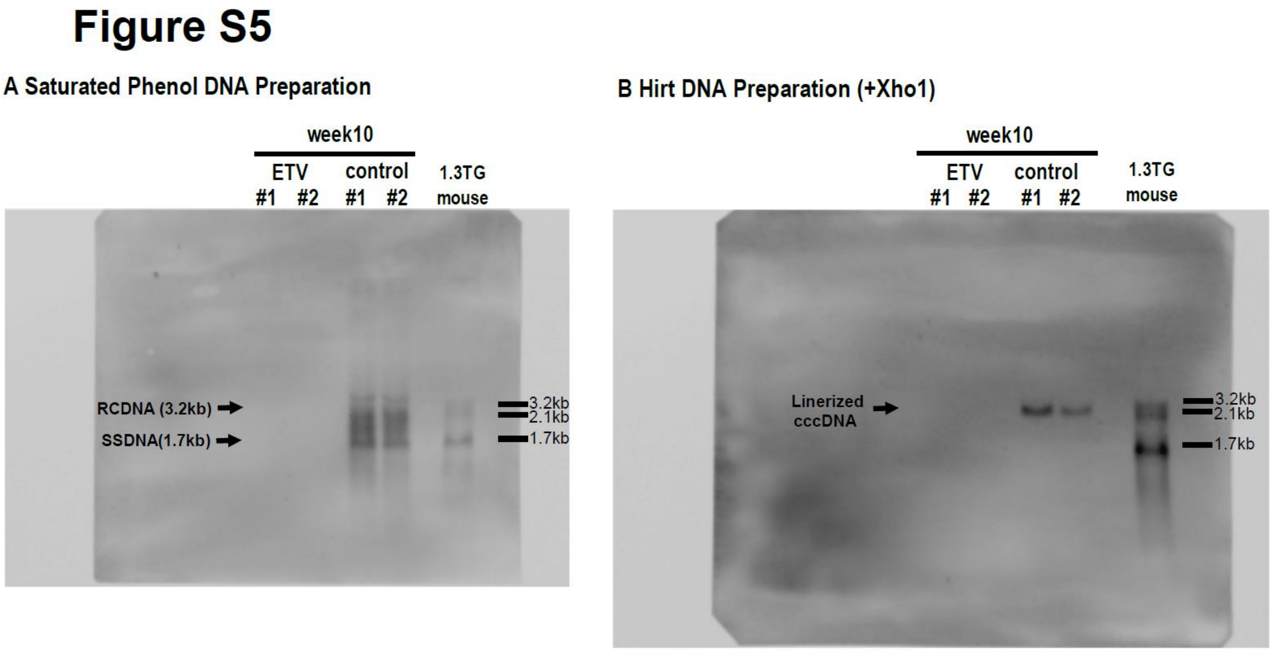

Figure S5. Kinetics of intrahepatic cccDNA in the chimeric mice during ETV treatment. Southern blot analysis of (A) HBV-DNA (rcDNA and ssDNA) and (B) cccDNA in the livers of HBV-infected chimeric mice, with or without ETV treatment, on week 10. The images of limited length were provided in Figure 4G and H.

Figure S6

A non-VR

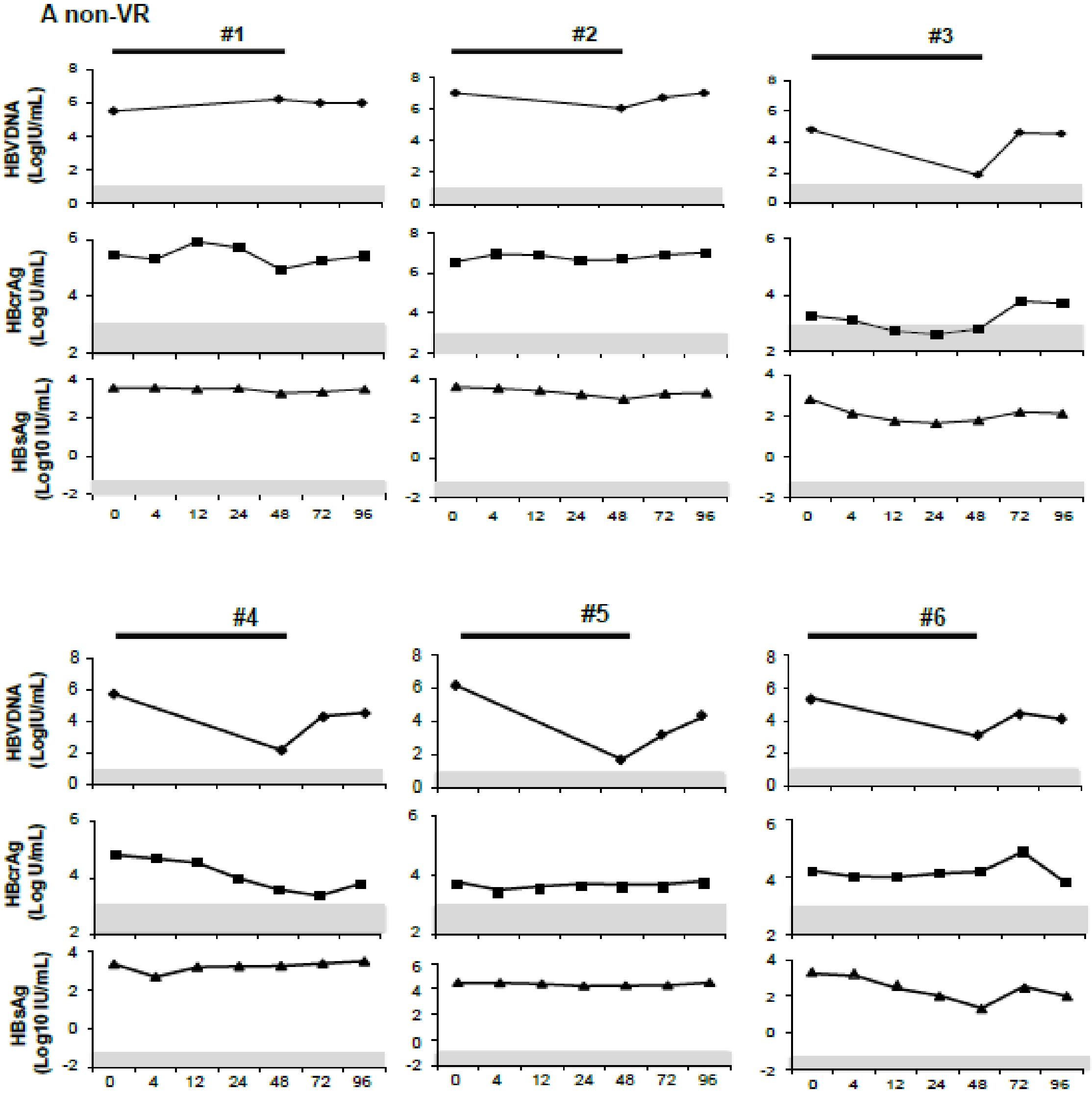

Figure S6

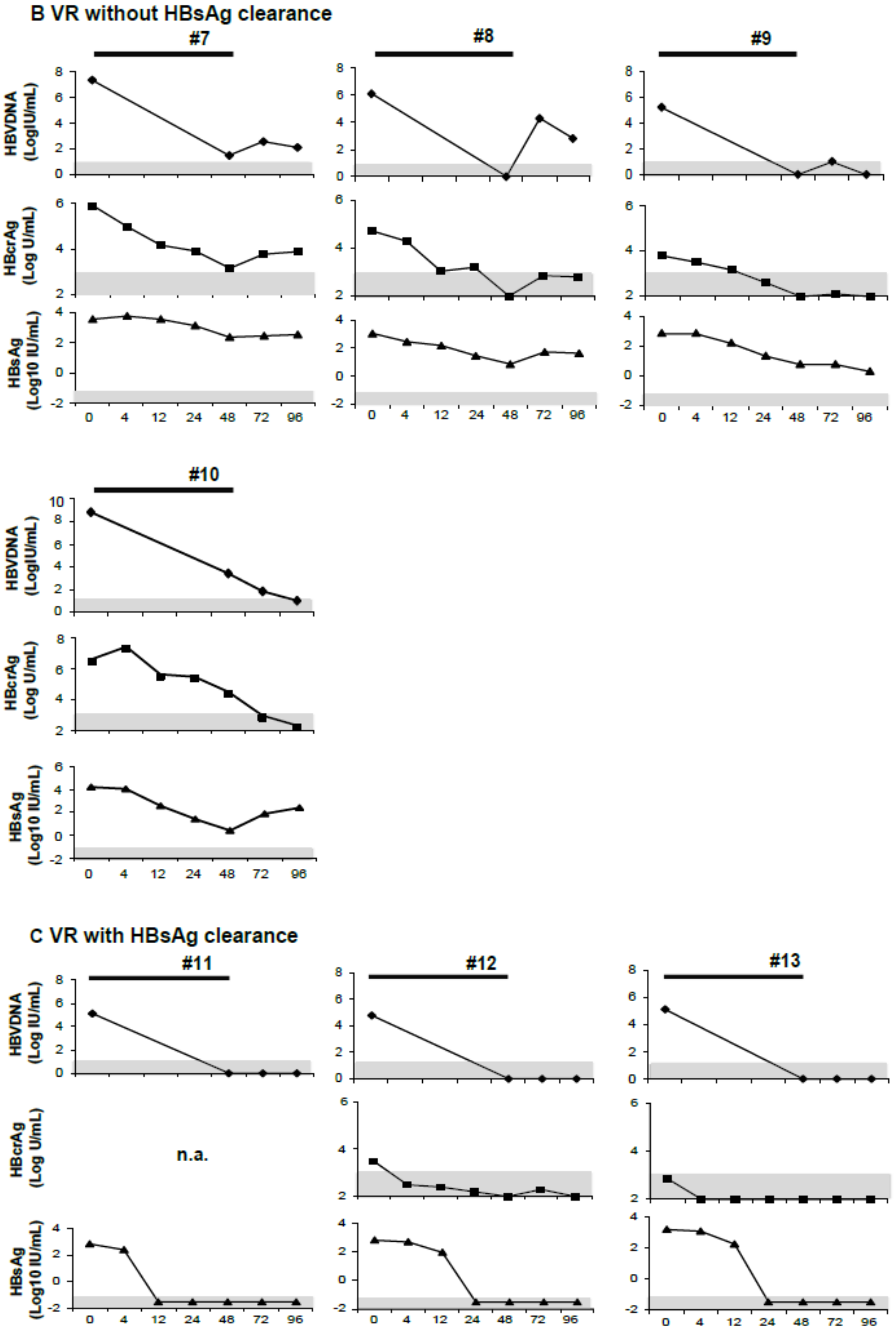
B VR without HBsAg clearance
#7
#8
#9
#10
HBVDNA (LogIU/mL)
HBcrAg (Log U/mL)
HBsAg (Log10 IU/mL)
0 4 12 24 48 72 96
C VR with HBsAg clearance
#11
#12
#13
HBVDNA (Log IU/mL)
HBcrAg (Log U/mL)
n.a.
HBsAg (Log10 IU/mL)

Figure S6. Kinetics of serum HBV DNA, HBcrAg and HBsAg levels in chronic hepatitis B patients during PEG-IFN treatment.

Changes in the levels of HBV DNA, HBcrAg and HBsAg in sera were determined in patients (A) with non-VR, (B) VR without HBsAg clearance and (C) VR with HBsAg clearance, during and after PEG-IFN treatment. Preserved serum samples from one patient, #11, were not available.

Figure S7

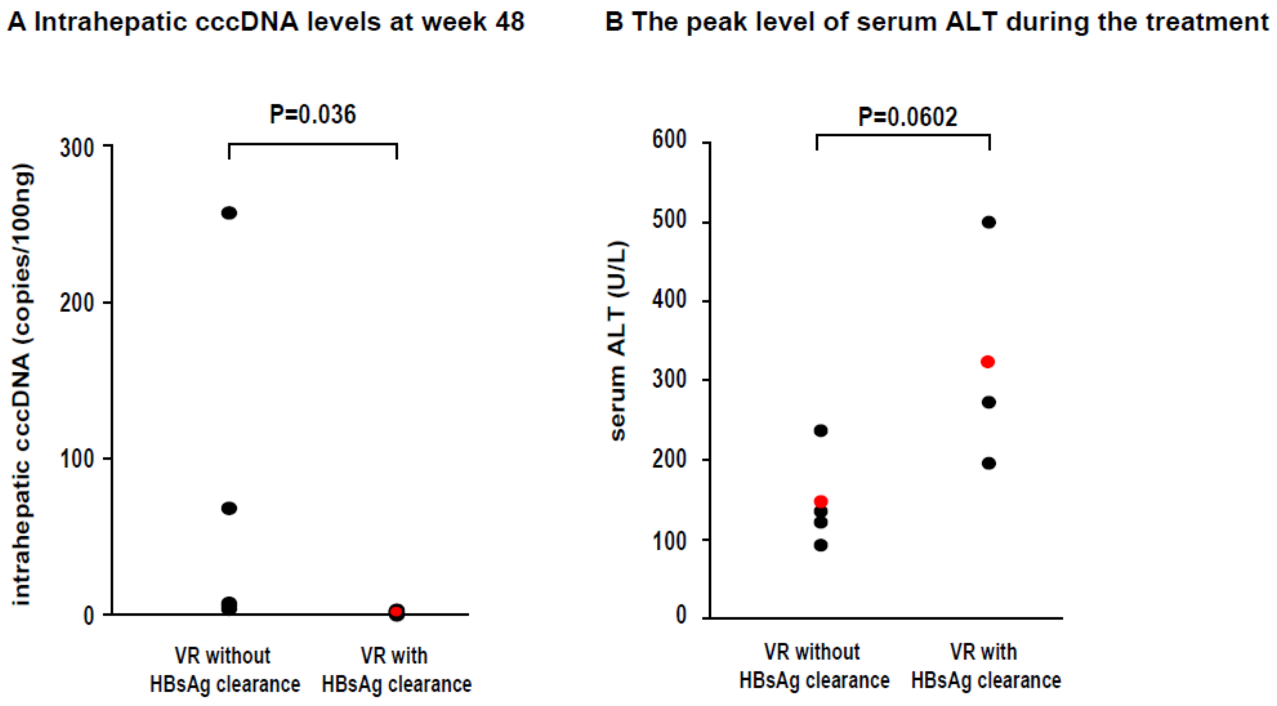

Figure S7. Comparison of intrahepatic cccDNA and serum ALT during PEG-IFN treatment in VR patients without or with HBsAg clearance.

The mean titers of intrahepatic cccDNA at week 48 were 68.4 (3.6-256.8) in VR without HBsAg clearance and 1.6 (0.4-3.3) in VR with HBsAg clearance (A). The peak titers of ALT during PEG-IFN treatment were 146.5 (93-237) in VR without HBsAg clearance and 322.7 (195-500) in VR with HBsAg clearance (B). Comparison between the groups was performed using the Mann-Whitney test.

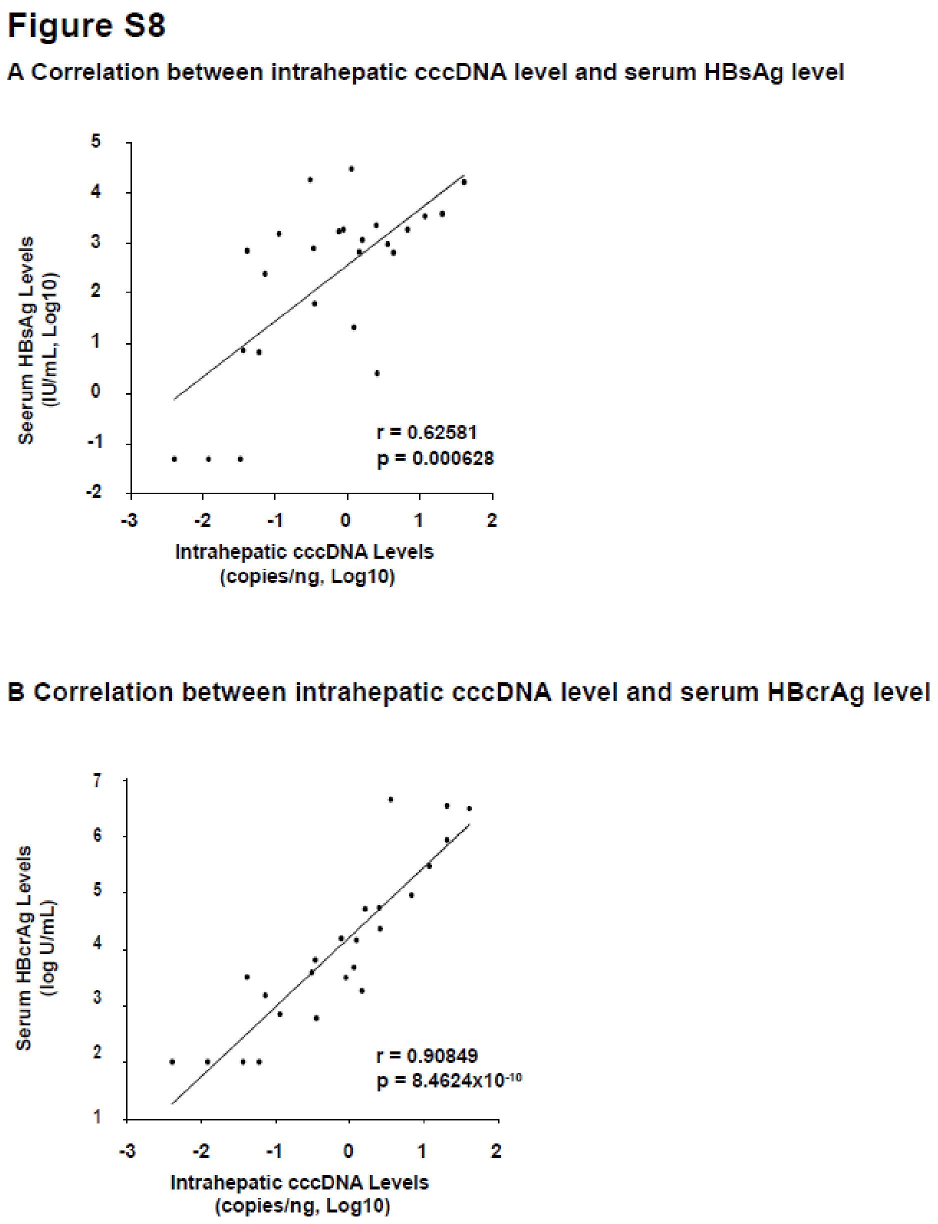

Figure S8. Correlation between intrahepatic cccDNA and serum HBV parameters.

The cccDNA contents of the livers of patients with HBeAg-negative CHB who received PEG-IFN therapy were measured and correlated with (A) serum HBsAg and (B) serum HBcrAg, at baseline and 48 weeks.

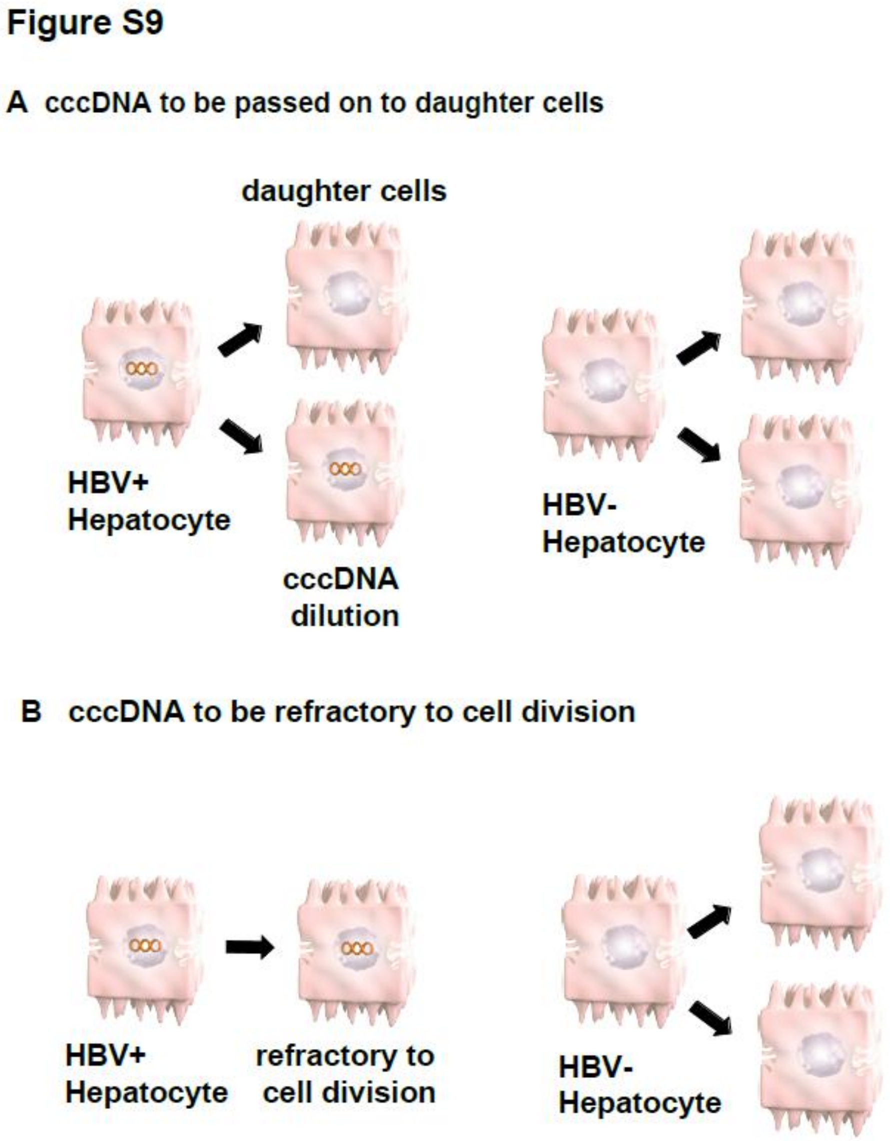

Figure S9. Persistence of intrahepatic cccDNA in dividing hepatocytes.

Our *in vivo* study indicated that the total amount of intrahepatic cccDNA did not reduce despite the number of hepatocytes doubling in the absence of active HBV replication. It is possible that (A) cccDNA survives hepatocyte mitosis and passes to a daughter cell or (B) HBV infected hepatocytes are unable to divide and the liver was mainly reconstituted by the

proliferation of uninfected hepatocytes.
